# Author Correction: Molecular patterns identify distinct subclasses of myeloid neoplasia

**DOI:** 10.1038/s41467-024-45644-x

**Published:** 2024-02-08

**Authors:** Tariq Kewan, Arda Durmaz, Waled Bahaj, Carmelo Gurnari, Laila Terkawi, Hussein Awada, Olisaemeka D. Ogbue, Ramsha Ahmed, Simona Pagliuca, Hassan Awada, Yasuo Kubota, Minako Mori, Ben Ponvilawan, Bayan Al-Share, Bhumika J. Patel, Hetty E. Carraway, Jacob Scott, Suresh K. Balasubramanian, Taha Bat, Yazan Madanat, Mikkael A. Sekeres, Torsten Haferlach, Valeria Visconte, Jaroslaw P. Maciejewski

**Affiliations:** 1 Department of Translational Hematology and Oncology Research, Taussig Cancer Institute, Cleveland Clinic, Cleveland, OH USA; 2 Department of Hematology and Medical Oncology, Yale University, New Haven, CT USA; 3 Systems Biology and Bioinformatics Department, School of Medicine, Case Western Reserve University, Cleveland, OH USA; 4 Department of Biomedicine and Prevention, Ph.D. in Immunology, Molecular Medicine and Applied Biotechnology, University of Rome Tor Vergata, Rome, Italy; 5 Department of Clinical Hematology, CHRU de Nancy, Nancy, France; 6 Roswell Park Comprehensive Cancer Center, Buffalo, NY USA; 7 Department of Hematology and Oncology, Karmanos Cancer Institute, Wayne State University, Detroit, MI USA; 8 Department of Internal Medicine, Division of Hematology and Oncology, University of Texas Southwestern Medical Center, Dallas, TX USA; 9 Division of Hematology, Sylvester Cancer Center, University of Miami, Miami, FL USA; 10 MLL Munich Leukemia Laboratory, Munich, Germany

**Keywords:** Myelodysplastic syndrome, Cancer epigenetics, Acute myeloid leukaemia

Correction to: *Nature Communications* 10.1038/s41467-023-38515-4, published online 30 May 2023

In this article the author name Yasuo Kubota was incorrectly written as Yasuo Kutoba. The original article has been corrected.

