# Supplementary material for: Molecular patterns identify distinct subclasses of myeloid neoplasia
Source: Nat Commun. 2023 May 30;14:3136. doi: 10.1038/s41467-023-38515-4 (PMC10229666; doi:10.1038/s41467-023-38515-4)
Supplement: Supplementary file 1 — Supplementary Information [file 41467_2023_38515_MOESM1_ESM.pdf]

Supplemental materials

Molecular patterns identify distinct subclasses of myeloid neoplasia

Content:

A) Supplementary tables

- **Supplementary Table-1.** Summary of all the sources of cases included in our study.
- **Supplementary Table-2.** Summary of clinical and molecular features of all cohorts.
- **Supplementary Table-3.** List of 40 genes used in our molecular clustering scheme.
- **Supplementary Table-4.** Clinical, cytogenetic, and molecular features of experimental and validation cohorts.
- **Supplementary Table-5 and Table-6.** Clinical, cytogenetic, and molecular features of original and validation cohorts based on clusters.
- **Supplementary Table-7.** Molecular signatures of all novel clusters.
- **Supplementary Table-8.** Median overall survival of the validation cohort based on the assigned clusters.
- **Supplementary Table-9.** Clinical, cytogenetic, and molecular features of all risk groups.
- **Supplementary Table-10.** Hazard risk (HR) coefficients of the identified risk groups across the training and the validation datasets.
- **Supplementary Table-11.** Hazard risk (HR) coefficients of the identified risk groups adjusted for IPSS-R.
- **Supplementary Table-12.** Hazard risk (HR) coefficients of the identified risk groups adjusted for IPSS-M.

B) Supplementary figures

- **Supplementary Figure 1.** The frequency of total mutations numbers.
- **Supplementary Figure 2.** The frequency of genetic mutations and cytogenetic abnormalities based on genomic clusters.
- **Supplementary Figure 3.** The most important genetic features used in our scheme.
- **Supplementary Figure 4.** The most important genetic features used in our scheme at the level of each cluster.
- **Supplementary Figure 5.** K-fold cross validation method.
- **Supplementary Figure 6.** Clustering based on BayesLCA model as a baseline model comparison.
- **Supplementary Figure 7.** Clinical characteristics of our molecular clusters.

- **Supplementary Figure 8.** The distribution of genomic clusters among patients with different clinical diagnosis.
- **Supplementary Figure 9.** The median bone marrow blast percent per genomic clusters.
- **Supplementary Figure 10.** Genomic clusters percentage within all the molecular mutations and cytogenetic abnormalities.
- **Supplementary Figure 11.** Kaplan-Meier analysis showing the overall survival (in months) of cases assigned to different clusters.
- **Supplementary Figure 12.** Kaplan-Meier analysis showing the overall survival (in months) for training set and the validation cohort based on the assigned molecular clusters.
- **Supplementary Figure 13.** Cox proportional hazard model with clinical features for overall survival
- **Supplementary Figure 14.** Kaplan-Meier analysis showing the overall survival (in months) of MDS cases based on IPSS-R subgroups.
- **Supplementary Figure 15.** Kaplan-Meier analysis showing the overall survival (in months) of MDS cases based on IPSS-M subgroups
- **Supplementary Figure 16.** Bootstrapped distribution of C-index differences between our model and IPSS-M.
- **Supplementary Figure 17.** Molecular International Prognostic Scoring System (IPSS-M) scores in our molecular clusters.

C) Supplementary figure legends.

D) Supplementary methods.

Genetic studies

- **Conventional cytogenetics**
- **Statistical Methods**
- **Autoencoder**
- **Gaussian Mixture Model**
- **Unsupervised clustering**
- **Classification of New Data**
- **Validation**

E) Supplementary Notes.

F) References.

Supplementary Table-1. Summary of the sources of myelodysplastic syndrome and secondary acute myeloid leukemia cases included in our study

Cohorts	Total number of patients
**Our cohorts**	2902
Cleveland Clinic Foundation (CCF)	1627
Munich Leukemia Laboratory (MLL)	1275
**Public cohorts#**	686
Beat AML master trial[1]	45
Euro-MDS cohort[2]	641
**External Validation Cohorts**	419
Wayne State University Karmanos Comprehensive cancer center	207
University of Texas Southwestern Simmons Comprehensive Cancer Center	212

Only cases with available cytogenetics and molecular features were included.

1 Tyner JW, Tognon CE, Bottomly D, et al. Functional genomic landscape of acute myeloid leukaemia. Nature 2018;562:526-31.

2 Bersanelli M, Travaglino E, Meggendorfer M, et al. Classification and Personalized Prognostic Assessment on the Basis of Clinical and Genomic Features in Myelodysplastic Syndromes. J Clin Oncol 2021;39:1223-33.

Supplementary Table-2. Clinical, cytogenetic, and molecular characteristics of original cohorts

Variables	Cleveland Clinic Foundation (CCF) **N=1627**	Munich Leukemia Laboratory (MLL) **N= 1275**	Beat AML master trial **N=45**	Euro-MDS cohort **N=641**
**Age, median (IQR)**	69 (61-76)	73 (67-78)	69 (62-74)	74 (68-79)
**Gender**				
Male, n (%)	59	60	69	61
Female, n (%)	41	40	31	39
**BM blast %, median (IQR)**	3 (1-13)	6 (2-20)	37 (21-62)	4 (2-7)
**Diagnosis**				
LR-MDS	1041 (64)	611 (48)	0	427 (67)
HR-MDS	277 (17)	283 (22)	0	214 (33)
s-AML	309 (19)	381 (30)	45	0
**Cytogenetics**				
Normal	774 (48)	757 (59)	17 (38)	475 (74)
Abnormal	837 (52)	517 (41)	28 (62)	166 (26)
Complex	287 (18)	66 (5)	11 (24)	26 (4)
**Molecular Clusters**				
1	79 (5)	98 (8)	3 (7)	21 (3)
2	436 (27)	325 (26)	17 (38)	142 (22)
3	35 (2)	29 (2)	0 (0)	12 (2)
4	74 (5)	125 (10)	0 (0)	114 (18)
5	71 (4)	25 (2)	0 (0)	11 (2)
6	98 (6)	114 (9)	0 (0)	89 (14)
7	166 (10)	24 (2)	0 (0)	35 (5)
8	69 (4)	131 (10)	1 (2)	35 (5)
9	88 (5)	89 (7)	0 (0)	42 (7)
10	31 (2)	49 (4)	0 (0)	63 (10)
11	70 (4)	43 (3)	2 (5)	6 (1)
12	49 (3)	56 (4)	0	25 (4)
13	287 (18)	67 (5)	11 (24)	26 (4)
14	74 (5)	100 (8)	11 (24)	20 (3)
**Status Death, n (%)**	752 (46)	521 (41)	31 (76)	255 (40)

IQR: interquartile range, BM, bone marrow, LR, low risk; HR, high risk, sAML, secondary acute myeloid leukemia. No statistical test was applied.

Supplementary Table-3. List of 40 genes in our targeted panel used for the molecular machine learning model

ASXL1	BCOR	BCORL1	CALR	CUX1	CEBPA	CBL	CSF3R
DNMT3A	DDX41	EZH2	ETV6	FLT3	GATA2	GNAS	IDH1
IDH2	JAK2	KRAS	KIT	MPL	NRAS	NPM1	NF1
NOTCH1	PTPN11	PHF6	RAD21	RUNX1	SF3B1	SRSF2	SMC1A
SMC3	STAG2	SETBP1	TET2	TP53	U2AF1	WT1	ZRSR2

Supplementary Table-4. Clinical, cytogenetic, and molecular characteristics of original and validation cohorts

Variables	Experimental Cohort N=3588	External Cohort N= 412	P-value
**Age, median (IQR)**	72 (64-77)	69 (62-75)	0.000
**Gender**			0.815
Male, n (%)	2143 (60)	248 (60)	
Female, n (%)	1444 (40)	163 (40)	
**BM blast %, median (IQR)**	4 (2-13)	7 (2-19)	0.000
**Diagnosis**			0.000
LR-MDS	2079 (58)	156 (38)	
HR-MDS	774 (22)	134 (33)	
s-AML	735 (20)	122 (30)	
**Cytogenetics**			0.000
Normal	2023 (57)	252 (62)	
Abnormal	1548 (43)	156 (38)	
**Number of MT**			0.000
0	825 (23)	33 (8)	
1-2	1666 (46)	204 (49)	
3-4	813 (23)	114 (28)	
>4	581 (16)	61 (15)	
**Molecular clusters**			
1	201 (6)	25 (6)	
2	920 (26)	74 (18)	
3	76 (2)	2 (1)	
4	313 (9)	17 (4)	
5	107 (3)	10 (2)	
6	301 (8)	19 (5)	
7	225 (6)	54 (13)	
8	236 (7)	13 (4)	
9	219 (6)	26 (6)	
10	143 (4)	9 (2)	
11	121 (3)	16 (4)	
12	130 (4)	19 (5)	
13	391 (11)	118 (29)	
14	205 (6)	10 (2)	
**Status Death, n (%)**	1559 (44)	190 (46)	0.315

IQR: interquartile range, MDS: myelodysplastic syndrome, sAML: secondary acute myeloid leukemia, LR: low-risk, HR: high-risk, MT: mutation, BM: bone marrow. Statistics were calculated with a two-sided nonparametric Wilcoxon matched-pairs signed rank test for numerical variables and Chi square test for categorical variables, p-values are reported in the table.

Supplementary Table-5. Clinical, cytogenetic, and molecular characteristics of original and validation cohorts clusters C1-C7

	C1			C2			C3			C4			C5			C6			C7		
**Variables**	**O**	**V**	**P**	**O**	**V**	**P**	**O**	**V**	**P**	**O**	**V**	**P**	**O**	**V**	**P**	**O**	**V**	**P**	**O**	**V**	**P**
	201	25		920	74		76	2		313	17		107	10		301	19		225	54	
**Age, M (IQR)**																					
**Gender**			0.779			0.866			0.467			0.368			0.031			0.391			0.921
Male	131 (65)	17 (68)		519 (56)	41 (55)		60 (79)	2 (100)		182 (58)	8 (47)		72 (67)	10 (100)		203 (67)	11 (58)		135 (60)	32 (59)	
Female	70 (44)	8 (32)		401 (44)	33 (45)		16 (21)	0 (0)		131 (42)	9 (53)		35 (33)	0 (0)		35 (33)	8 (42)		90 (40)	22 (41)	
**BM blast %**																					
**Diagnosis**			0.004			0.035			0.358			0.009			0.787			0.117			0.000
LR-MDS	73 (36)	4 (16)		566 (62)	35 (47)		37 (49)	2 (100)		268 (86)	10 (59)		77 (72)	7 (70)		152 (51)	9 (47)		147 (65)	23 (43)	
HR-MDS	50 (25)	14 (56)		148 (16)	19 (26)		28 (37)	0 (0)		27 (9)	5 (29)		14 (13)	2 (20)		116 (39)	5 (26)		49 (22)	10 (19)	
sAML	78 (39)	7 (28)		206 (22)	20 (27)		11 (15)	0 (0)		18 (6)	2 (12)		16 (15)	1 (10)		33 (11)	5 (26)		29 (13)	21 (39)	
**Cytogenetics**						0.000			-			-			-			0.008			
Normal	1 (1)	0 (0)		920 (100)	71 (96)		0 (0)	0 (0)		313 (100)	17 (100)		0 (0)	0 (0)		300 (99.7)	18 (95)		0 (0)	0 (0)	
Abnormal	200 (100)	25 (100)		0 (0)	3 (4)		76 (100)	2 (100)		0 (0)	0 (0)		106 (100)	10 (100)		1 (0.3)	1 (5)		221 (100)	54 (100)	
**MT #**			0.015			0.000			0.952			0.000			0.775			0.000			0.066
0	40 (20)	0 (0)		446 (49)	16 (22)		0 (0)	0 (0)		0 (0)	0 (0)		22 (21)	2 (20)		0 (0)	0 (0)		39 (17)	1 (2)	
1-2	70 (35)	11 (44)		366 (40)	26 (49)		45 (59)	1 (50)		235 (75)	9 (53)		55 (51)	5 (50)		162 (54)	7 (37)		109 (48)	27 (50)	
3-4	57 (28)	8 (32)		77 (8)	25 (34)		26 (34)	1 (50)		68 (22)	6 (36)		21 (20)	2 (20)		117 (39)	7 (37)		50 (22)	18 (34)	
>4	34 (17)	6 (24)		31 (3)	7 (9)		5 (7)	0 (0)		10 (13)	2 (12)		9 (8)	1 (10)		22 (7)	5 (26)		27 (12)	8 (19)	

IQR: interquartile range, MDS: myelodysplastic syndrome, sAML: secondary acute myeloid leukemia, LR: low-risk, HR: high-risk, MT: mutation, #: number, BM: bone marrow, C: cluster, O: original cohort, V: validation cohort, P:p-value. Statistics were calculated with a two-sided nonparametric Wilcoxon matched-pairs signed rank test for numerical variables and Chi square test for categorical variables, p-values are reported in the table.

Supplementary Table-6. Clinical, cytogenetic, and molecular characteristics of original and validation cohorts clusters C8-C14

		C8			C9			C10			C11			C12			C13			C14	
**Variables**	**O**	**V**	**P**	**O**	**V**	**P**	**O**	**V**	**P**	**O**	**V**	**P**	**O**	**V**	**P**	**O**	**V**	**P**	**O**	**V**	**P**
	236	13		219	26		143	9		121	16		130	19		391	117		205	10	
**Age, M (IQR)**																					
**Gender**			0.523			0.480			0.888			0.903			0.289			0.506			0.722
Male	71 (30)	5 (38)		163 (74)	21 (81)		76 (53)	5 (56)		70 (58)	9 (56)		91 (70)	11 (58)		217 (56)	69 (59)		153 (75)	7 (70)	
Female	165 (70)	8 (62)		56 (26)	5 (19)		67 (47)	4 (44)		51 (42)	7 (44)		39 (30)	8 (42)		174 (45)	48 (41)		51 (25)	3 (30)	
**BM blast %**																					
**Diagnosis**			0.002			0.424			0.223			0.057			0.476			0.012			0.161
LR-MDS	184 (78)	6 (46)		96 (44)	8 (31)		112 (78)	8 (89)		46 (38)	3 (19)		68 (52)	8 (42)		150 (38)	28 (24)		103 (50)	5 (50)	
HR-MDS	21 (9)	5 (38)		86 (39)	12 (46)		26 (18)	0 (0)		33 (27)	9 (56)		42 (32)	6 (32)		111 (28)	44 (37)		23 (11)	3 (30)	
sAML	31 (13)	2 (15)		37 (17)	6 (23)		5 (4)	1 (11)		42 (35)	4 (25)		20 (15)	5 (26)		130 (33)	46 (39)		79 (39)	2 (20)	
**Cytogenetics**			-			0.000			0.801			-			0.009			-			-
Normal	0 (0)	0 (0)		217 (99)	23 (89)		142 (99)	9 (100)		0 (0)	0 (0)		130 (100)	18 (95)		0 (0)	0 (0)		0 (0)	0 (0)	
Abnormal	236 (100)	13 (100)		2 (1)	3 (12)		1 (1)	0 (0)		121 (100)	16 (100)		0 (0)	1 (6)		391 (100)	117 (100)		205 (100)	10 (100)	
**MT #**			0.699			0.004			0.266			0.089			0.551			0.000			0.558
0	75 (32)	2 (15)		0 (0)	0 (0)		0 (0)	0 (0)		34 (28)	1 (6)		0 (0)	0 (0)		92 (24)	8 (7)		77 (38)	3 (30)	
1-2	130 (55)	11 (85)		57 (26)	3 (12)		63 (44)	1 (11)		54 (36)	7 (44)		7 (5)	0 (0)		219 (56)	80 (68)		104 (51)	6 (60)	
3-4	26 (11)	0 (0)		114 (52)	14 (54)		70 (49)	6 (67)		32 (26)	5 (31)		71 (55)	7 (37)		64 (16)	23 (20)		20 (10)	1 (10)	
>4	5 (2)	0 (0)		48 (22)	9 (35)		10 (7)	2 (22)		1 (1)	3 (19)		52 (40)	12 (63)		16 (4)	6 (5)		4 (2)	0 (0)	

IQR: interquartile range, MDS: myelodysplastic syndrome, sAML: secondary acute myeloid leukemia, LR: low-risk, HR: high-risk, MT: mutation, #: number, BM: bone marrow, C: cluster, O: original cohort, V: validation cohort, P:p-value. Statistics were calculated with a two-sided nonparametric Wilcoxon matched-pairs signed rank test for numerical variables and Chi square test for categorical variables, p-values are reported in the table.

Supplementary Table-7. Molecular clusters signatures

Risk Group	Clusters	Signature
**Group-1**	MC-4	Normal KT (100%), SF3B1 (100%), DNMT3A (20%)
	MC-10	Normal KT (100%), SF3B1 (100%), DNMT3A (24%), TET2 (100%)
**Group-2**	MC-2	Normal KT (100%), DNMT3A (11%), RAS MT (10%)
	MC-6	Normal KT (100%), SRSF2 (49%), RAS MT (23%)
	MC-8	Del 5q (100%), DNMT3A (17%), TP53 (17%)
**Group-3**	MC-3	Del Y (54%), TET2 (100%), ZRSR2 (23%), ASXL1 (21%)
	MC-5	Del 20q (76%), U2AF1 (28%), ASXL1 (16%)
	MC-7	Other KT (100%), Other MTs
	MC-9	Normal KT (100%), ASXL1 (100%), SRSF2 (34%), RUNX1 (31%)
	MC-12	Normal KT (100%), TET2 (100%), ASXL1 (100%), SRSF2 (48%), RUNX1 (40%)
	MC-14	Del Y (42%), SF3B1 (24%)
**Group-4**	MC-1	Trisomy 8 (100%), ASXL1 (35%), TET2 (31%), RUNX1 (24%)
	MC-11	Del 7q (100%), RAS MT (28%), TET2 (20%)
**Group-5**	MC-13	Complex (100%), TP53 (44%)

MC: molecular cluster, KT: karyotype, Del: deletion, MT: mutation

Supplementary Table-8. Median overall survival with 95% Confidence Intervals for the external validation cohort

Cluster	Total number	Median Survival
**Cluster-1**	25	16 (12-NA)
**Cluster-2**	75	NA (38-NA)
**Cluster-3**	2	5.5 (0-NA)
**Cluster-4**	17	NA (19-NA)
**Cluster-5**	10	NA (30-NA)
**Cluster-6**	19	30 (20-NA)
**Cluster-7**	54	47 (21-NA)
**Cluster-8**	13	36 (32-NA)
**Cluster-9**	26	26 (12-NA)
**Cluster-10**	9	NA (NA-NA)
**Cluster-11**	16	25 (13-NA)
**Cluster-12**	19	51 (11-NA)
**Cluster-13**	118	13 (8-28)
**Cluster-14**	10	NA (12-NA)

NA, not available

Supplementary Table-9. Clinical, cytogenetic, and molecular characteristics of all risk groups

Variables	All	Group-1	Group-2	Group-3	Group-4	Group-5
**Total population**	3588	456	1457	962	322	391
**Test cohort**	718 (20)	89 (19)	291 (20)	192 (20)	59 (18)	87 (22)
**Training cohort**	2870 (80)	367 (81)	1166 (80)	770 (80)	263 (82)	304 (78)
**Age, median (IQR)**	72 (64-77)	73 (68-78)	71 (63-77)	72 (64-78)	73 (65-78)	71 (63-77)
**Gender**						
**Male**	2143 (60)	258 (57)	793 (54)	674 (70)	201 (62)	217 (56)
**Female**	1444 (40)	198 (43)	664 (46)	287 (30)	121 (38)	174 (45)
**Labs**						
**WBC (10^9/L)**	4 (3-11)	6 (4-8)	5 (3-10)	6 (3-15)	5 (3-15)	3 (2-8)
**Hb(g/dL)**	10 (9-11)	10 (9-11)	10 (9-12)	10 (9-11)	9 (8-11)	10 (9-11)
**Platelets (10^9/L)**	112 (50-240)	290 (169-395)	112 (60-227)	102 (44-202)	67 (27-158)	58 (30-102)
**BM blast %**	4 (2-13)	2 (1-4)	4 (2-11)	5 (2-14)	11 (4-32)	12 (3-22)
**Diagnosis**						
**LR-MDS**	2079 (58)	380 (83)	902 (62)	528 (55)	119 (37)	150 (38)
**HR-MDS**	774 (22)	53 (12)	285 (20)	242 (25)	83 (26)	111 (28)
**sAML**	735 (21)	23 (5)	270 (19)	192 (20)	120 (37)	130 (33)
**Cytogenetics**						
**Normal**	2023 (57)	455 (100)	1220 (84)	347 (37)	1 (0)	0 (0)
**Abnormal**	1548 (43)	1 (0)	237 (16)	600 (63)	319 (100)	391 (100)
**Number of MT**						
**0**	825 (23)	0 (0)	521 (36)	138 (14)	74 (23)	92 (24)
**1-2**	1666 (46)	298 (66)	658 (45)	377 (39)	114 (35)	219 (56)
**3-4**	813 (23)	138 (31)	220 (16)	302 (31)	89 (28)	64 (17)
**>4**	284 (8)	20 (4)	58 (4)	145 (15)	45 (14)	16 (4)

IQR: interquartile range, WBC: white blood cell count, Hb: hemoglobin, MDS: myelodysplastic syndrome, sAML: secondary acute myeloid leukemia, LR: low-risk, HR: high-risk, MT: mutation, BM: bone marrow. No statistical comparison was performed.

Supplementary Table-10. Hazard-Risk (HR) Coefficients and associated CI and P-values comparing the aggregated risk groups to reference (Group-1) group across the training dataset and external validation dataset.

Risk Group	Training Set (HR, CI, SE, Z, P-Value)	Validation Set (HR, CI, SE, Z, P-Value)
Group-2	1.50 (1.22- 1.85, 0.10, 3.90, .001)	1.87 (0.79-4.24, 0.43, 1.43, 0.152)
Group-3	2.37 (1.92-2.93, 0.10, 8.00, <0.0001)	2.12 (0.91-4.92, 0.42, 1.75, 0.080)
Group-4	3.48 (2.71-4.46, 0.12, 9.81, <0.0001)	2.52 (1.01-6.27, 0.46, 1.98, 0.047)
Group-5	5.89 (4.68-7.41, 0.11, 15.1 <0.0001)	3.76 (1.63-8.69, 0.42, 3.10, 0.001)

Cox-Proportional Hazards Regression using risk groups as covariates. Statistics were calculated with cox proportional hazard model. Hazard ratio, P-values, and 95 % confidence intervals along with the test statistic from Wald Test and standard errors are reported in the table.

Supplementary Table-11. Hazard-Risk (HR) Coefficients and associated CI and P-values for our risk groups adjusted for IPSS-R and clinical variables. Time-dependent coefficients are used to account for proportionality assumption at 30 months cutoff (Time 1 < 30 months < Time 2)

Factor[#]	Training Set (HR, CI, SE, Z, P-Value)
Sex (Male)	1.35 (1.14-1.58, 0.0002, 0.08, 3.64)
log(BM+1.0)	0.98 (0.71-0.85, 0.814, 0.07, -0.23)
log(PLT+1.0)	0.78 (0.71-0.85, 1.10E-8, 0.04, -5.71)
HB	0.90 (86-95, 1.46E-5, 0.02, -4.33)
Group 2	1.23 (0.96-1.64, 0.1000, 0.12, 1.64)
Group 3	1.51 (1.16-1.98, 0.002, 0.13, 3.04)
Group 4	1.73 (1.01-2.46, 0.003, 0.18, 3.01)
Group 5	2.60 (1.81-3.75, 2.76E-7, 0.18, 5.13)
Age (Time 1)	1.02 (1.01-1.03, 9.27E-7, 0.004, 4.90)
Age (Time 2)	1.06 (1.04-1.08, 1.04E-12, 0.008, 7.12)
IPSSR Low (Time 1)	1.23 (0.84-1.81, 0.280, 0.19, 1.07)
IPSSR Low (Time 2)	0.87 (0.58-1.29, 0.478, 0.20, -0.70)
IPSSR Intermediate (Time 1)	1.64 (1.07-2.52, 0.022, 0.21, 2.29)
IPSSR Intermediate (Time 2)	0.86 (0.54-1.37, 0.517, 0.23, 0.64)
IPSSR High (Time 1)	2.91 (1.78-4.74, 1.84E-5, 0.24, 4.28)
IPSSR High (Time 2)	1.14 (0.63-2.08, 0.662, 0.30, 0.43)
IPSSR Very High (Time 1)	3.22 (1.80-5.79, 8.71E-5, 0.29, 3.92)
IPSSR Very High (Time 2)	1.97 (0.95-4.09, 0.069, 0.37, 1.82)

#: Statistics were calculated with adjusted cox proportional hazard model. Hazard risks, P-values, and 95 % confidence intervals are reported in the table. All the factors are compared to baseline which includes IPSS-R very low risk, Group1 risk group, mean of the continuous variables, and female sex.

Supplementary Table-12. Hazard-Risk (HR) Coefficients and associated CI and P-values for our risk groups adjusted for IPSS-M and clinical variables. Time-dependent coefficients are used to account for proportionality assumption at 30 months cutoff (Time 1 < 30 months < Time 2)

Factor[#]	Training Set (HR, CI, SE, Z, P-Value)
Sex (Male)	1.33 (1.14-1.56, 0.0002, 0.08, 3.56)
log(BM+1.0)	0.92 (0.81-1.05, 0.814, 0.06, -1.15)
log(PLT+1.0)	0.81 (0.74-0.89, 1.10E-8, 0.04, -4.44)
HB	0.94 (0.89-0.99, 1.46E-5,0.02, -2.29)
Group 2	1.18 (0.92-1.52, 0.100, 0.12, 1.33)
Group 3	1.32 (1.15-1.74, 0.002, 0.13, 2.02)
Group 4	1.63 (1.15-2.32, 0.003,0.17, 2.74)
Group 5	2.44 (1.72-3.44, 2.76E-7,0.17, 5.07)
Age (Time 1)	1.02 (1.01-1.03, 9.27E-7,0.004,4.70)
Age (Time 2)	1.06 (1.04-1.07, 1.04E-12,0.008,6.84)
IPSSM Low (Time 1)	1.28 (0.83-1.97, 0.259,0.22,1.12)
IPSSM Low (Time 2)	1.22 (0.80-1.84, 0.350, 0.21,0.93)
IPSSM Moderate Low (Time 1)	1.66 (1.00-2.77, 0.05, 0.25,1.96)
IPSSM Moderate Low (Time 2)	1.48 (0.86-2.55, 0.156,0.27,1.42)
IPSSM Moderate High (Time 1)	2.62 (1.57-4.35, 0.0002,0.25,3.72)
IPSSM Moderate High (Time 2)	1.21 (0.68-2.18, 0.508,0.29,0.66)
IPSSM High (Time 1)	3.63 (2.15-6.16, 1.52E-6,0.26,4.80)
IPSSM High (Time 2)	1.64 (0.88-3.06, 0.117,0.31,1.56)
IPSSM Very High (Time 1)	5.47 (3.02-9.89, 1.82E-8,0.30,5.62)
IPSSM Very High (Time 2)	1.69 (0.79-3.62, 0.174,0.38,1.35)

#: Statistics were calculated with adjusted cox proportional hazard model. Hazard risks, P-values, and 95 % confidence intervals are reported in the table. All the factors are compared to baseline which includes IPSS-M very low risk, Group1 risk group, mean of the continuous variables, and female sex.

Supplementary Figure-1

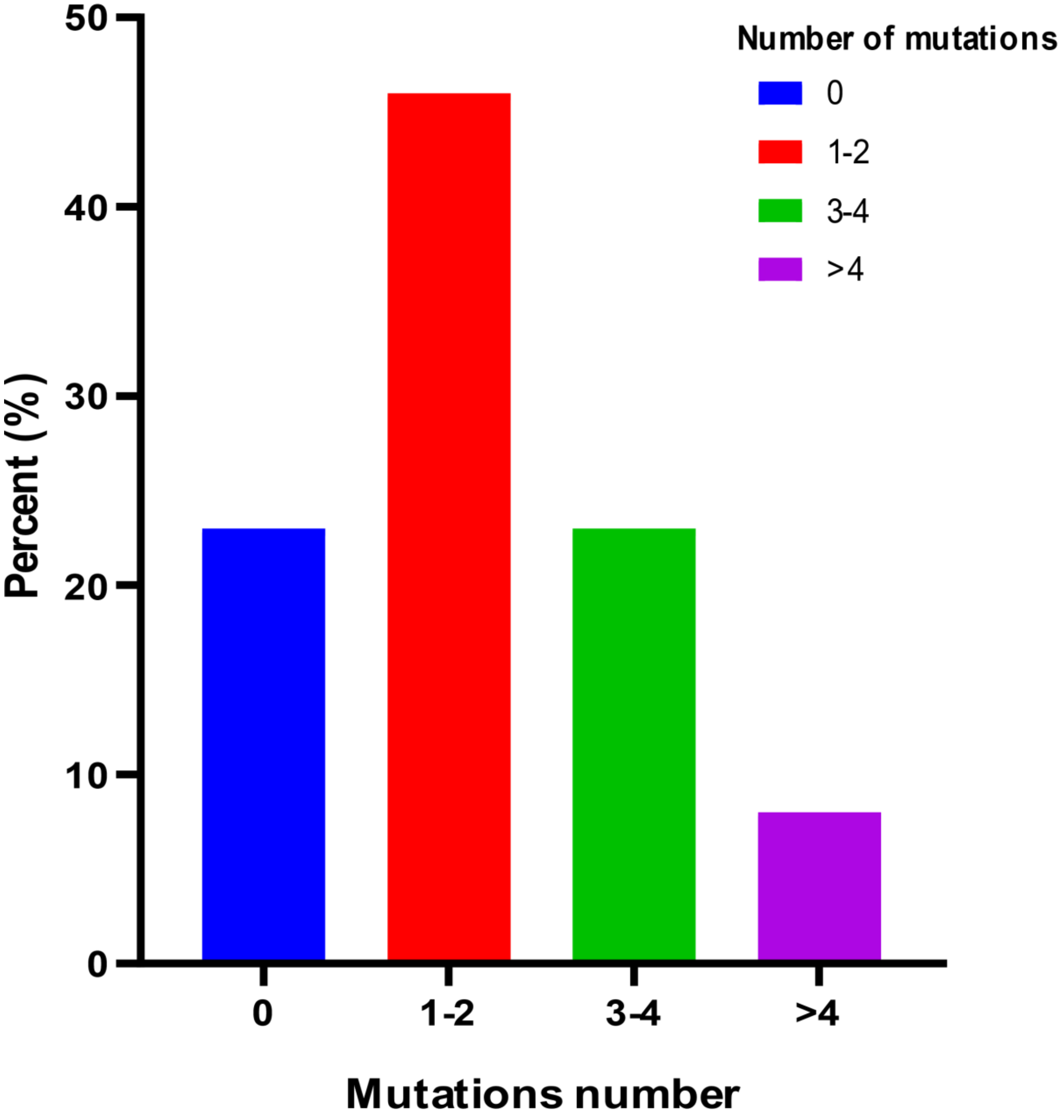

Supplementary Figure-2

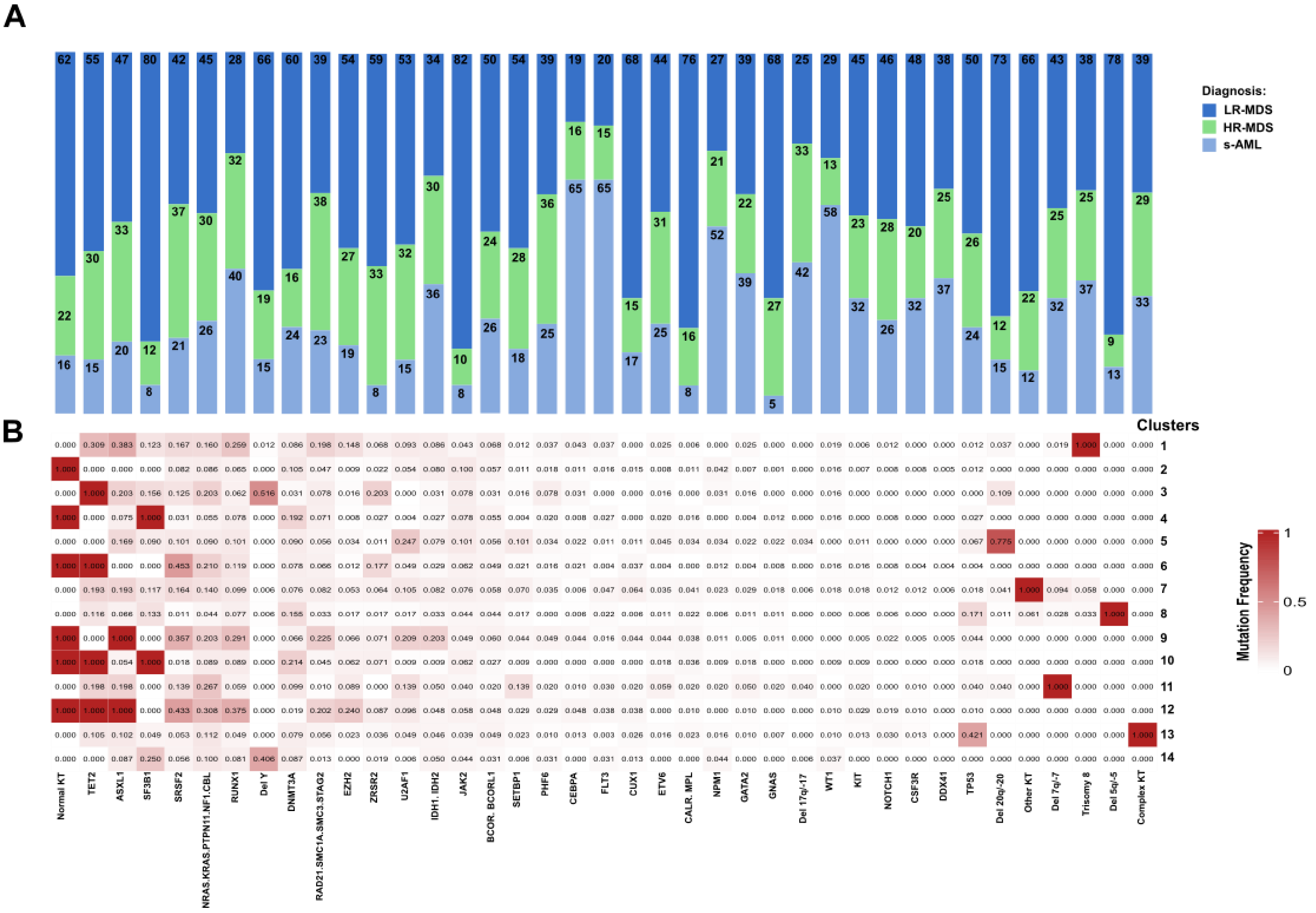

Supplementary Figure-3

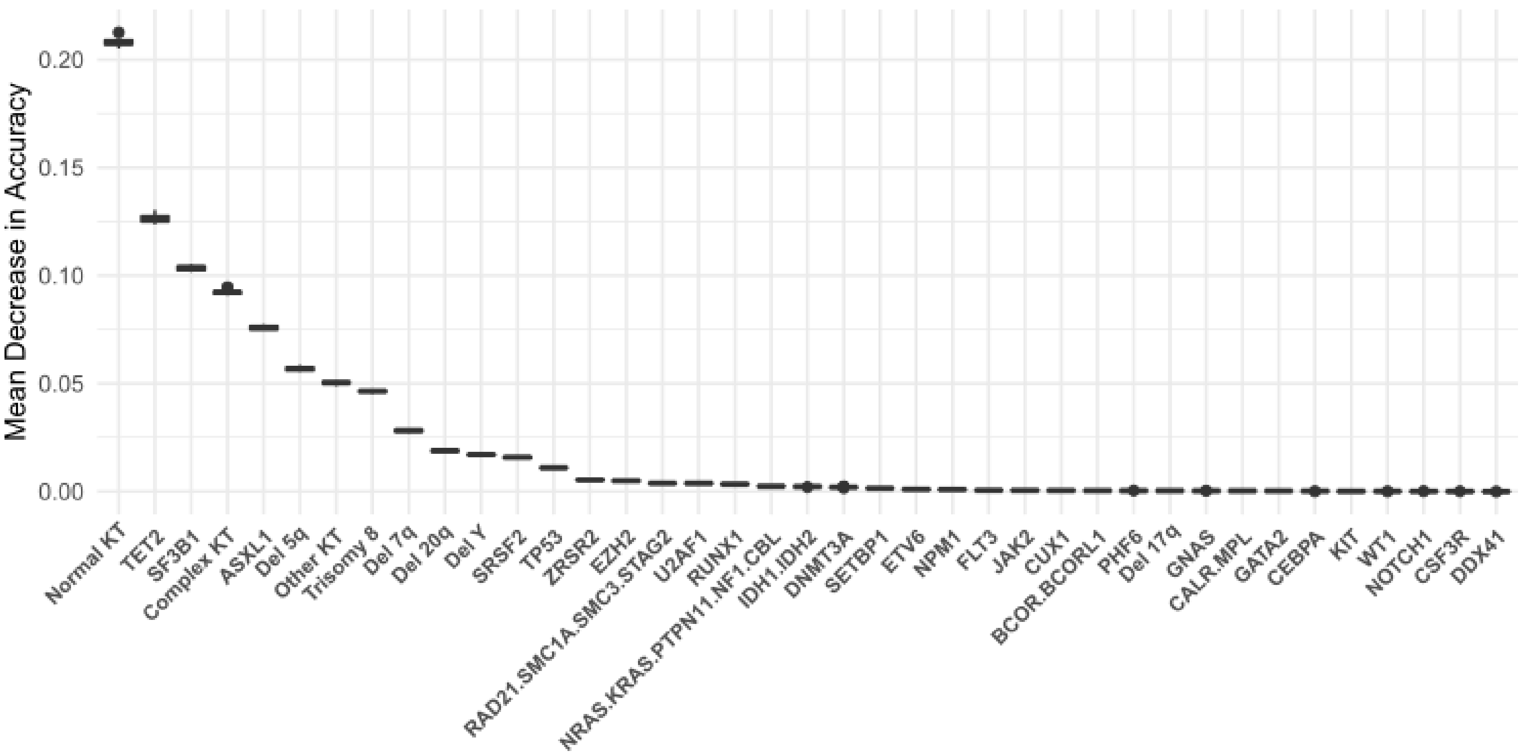

Supplementary Figure-4

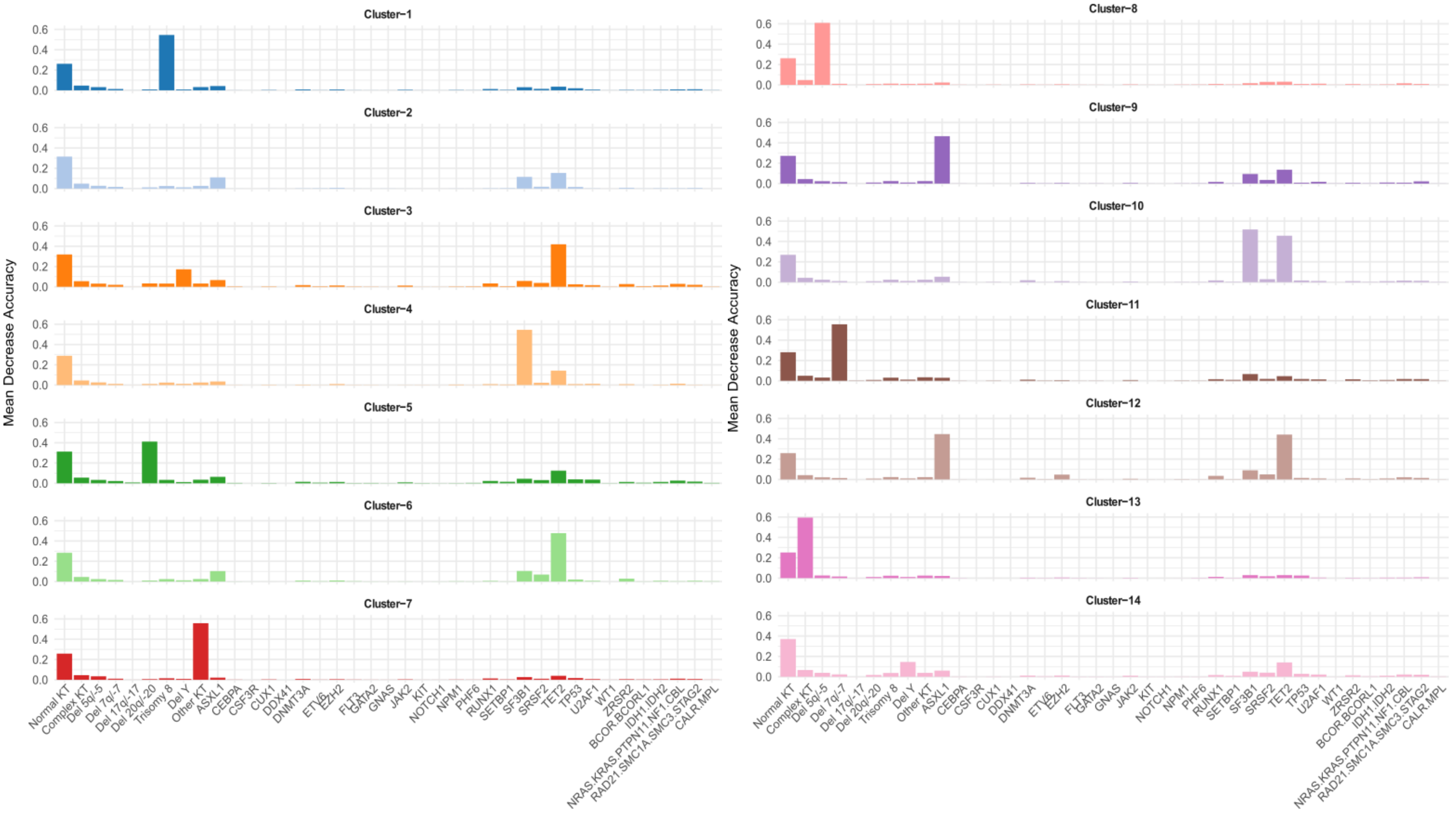

Supplementary Figure-5

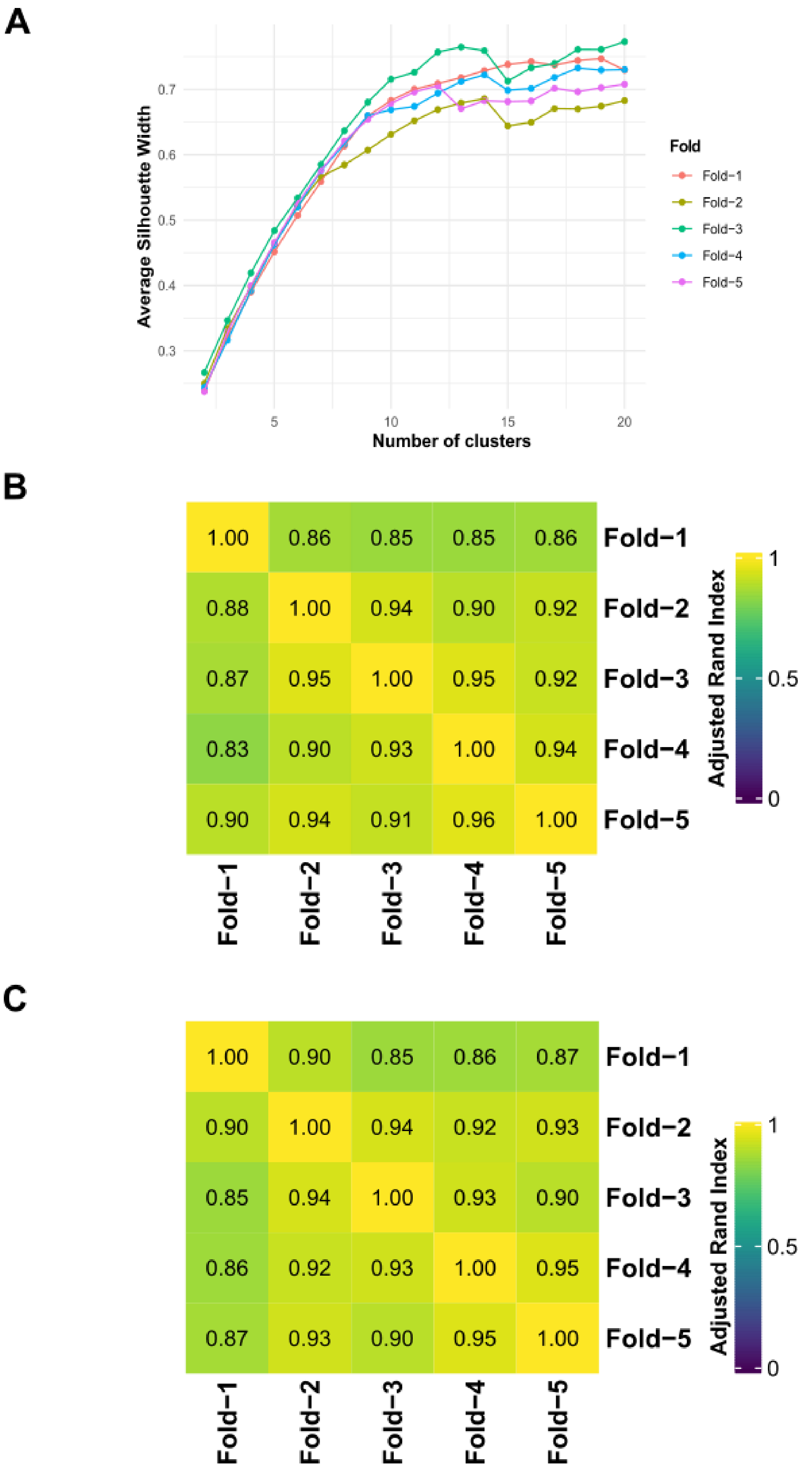

Supplementary Figure-6

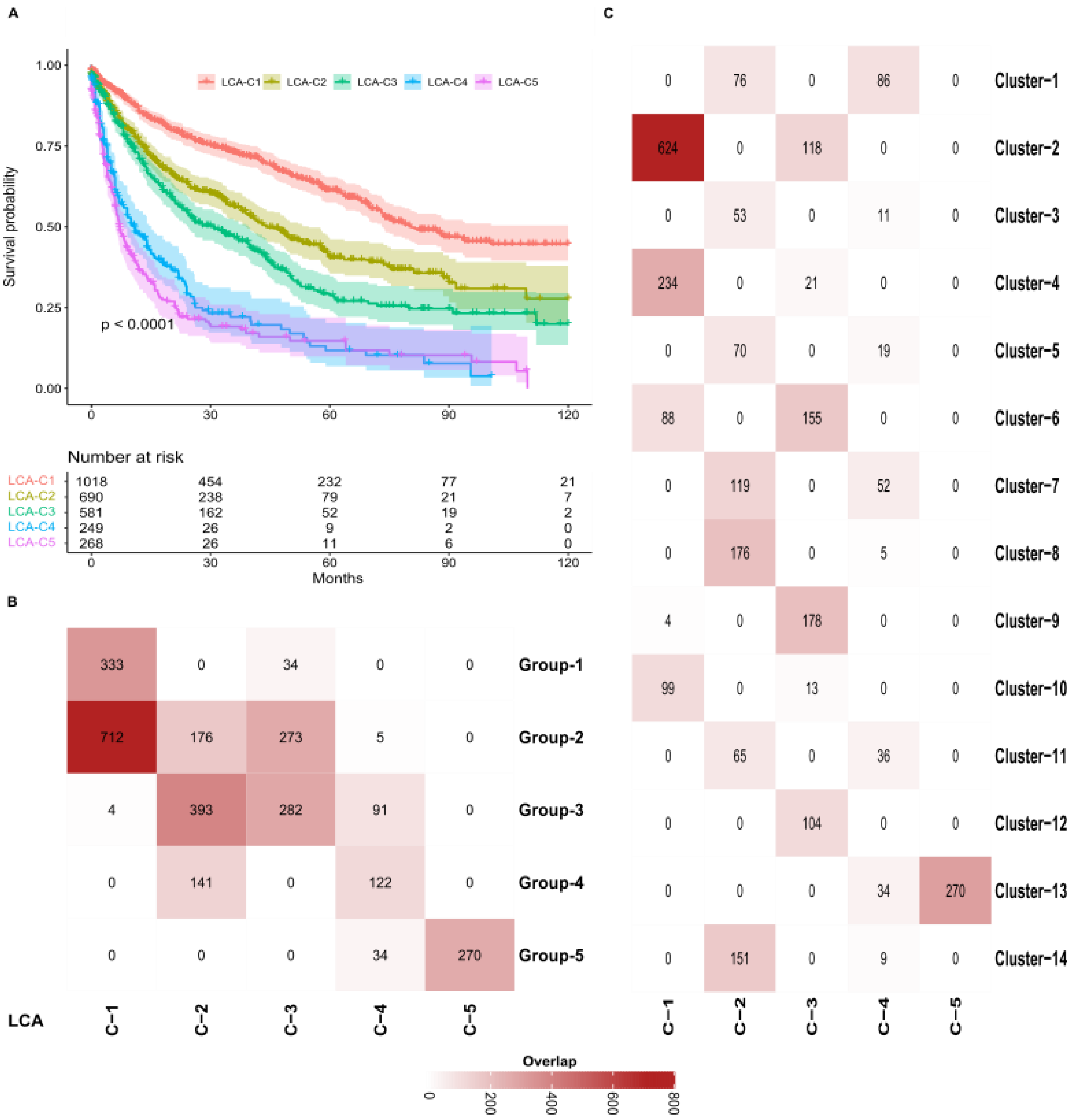

Supplementary Figure-7

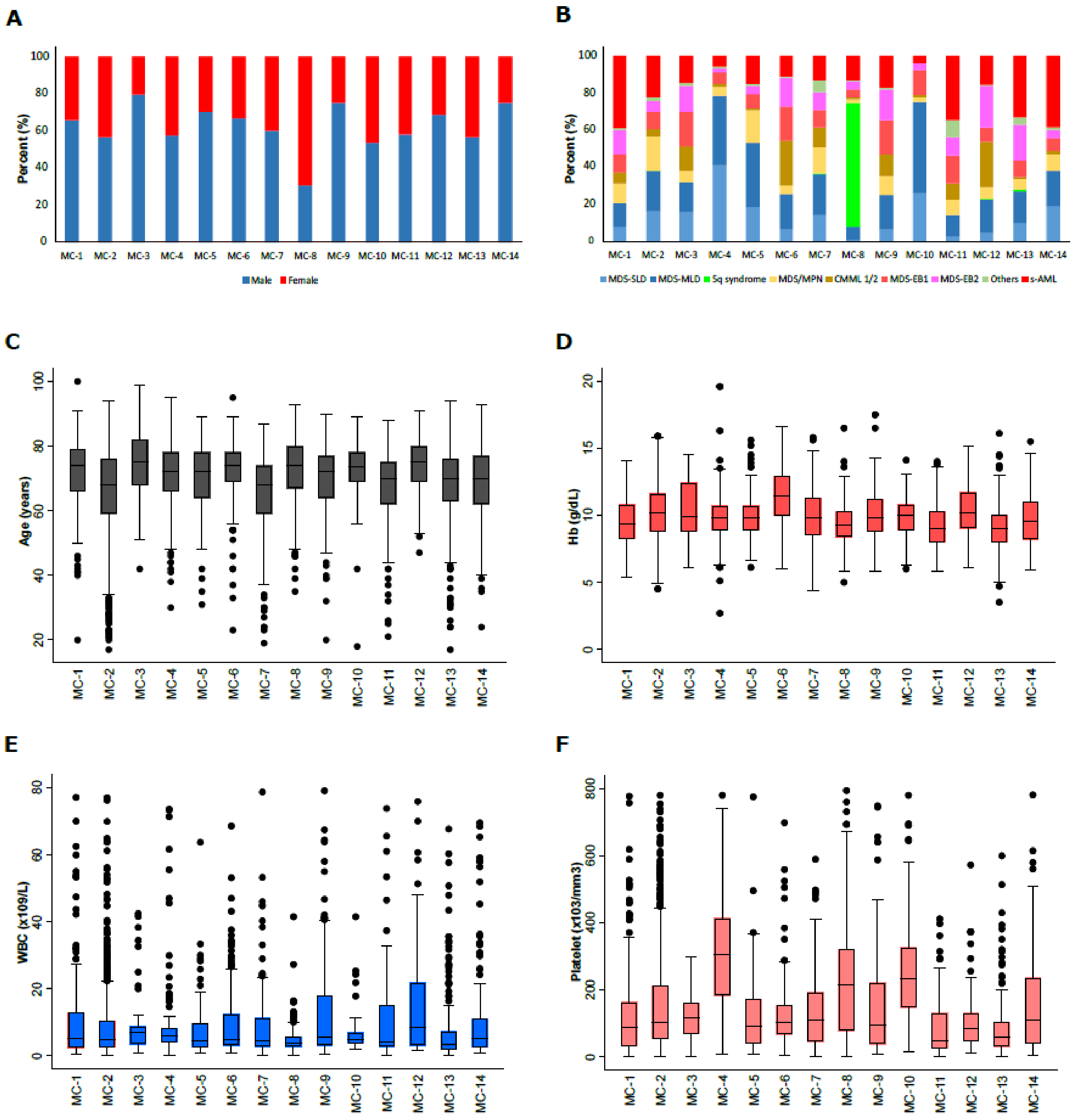

Supplementary Figure-8

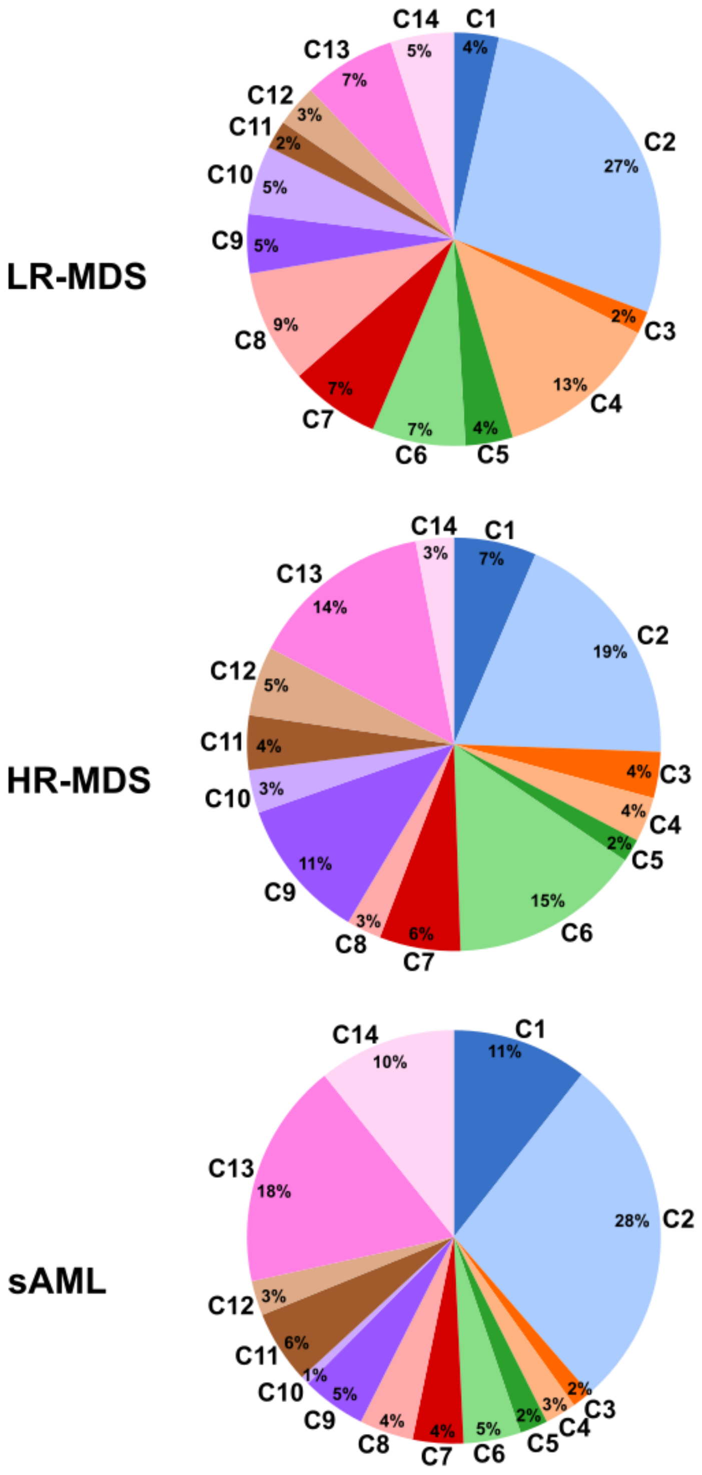

Supplementary Figure-9

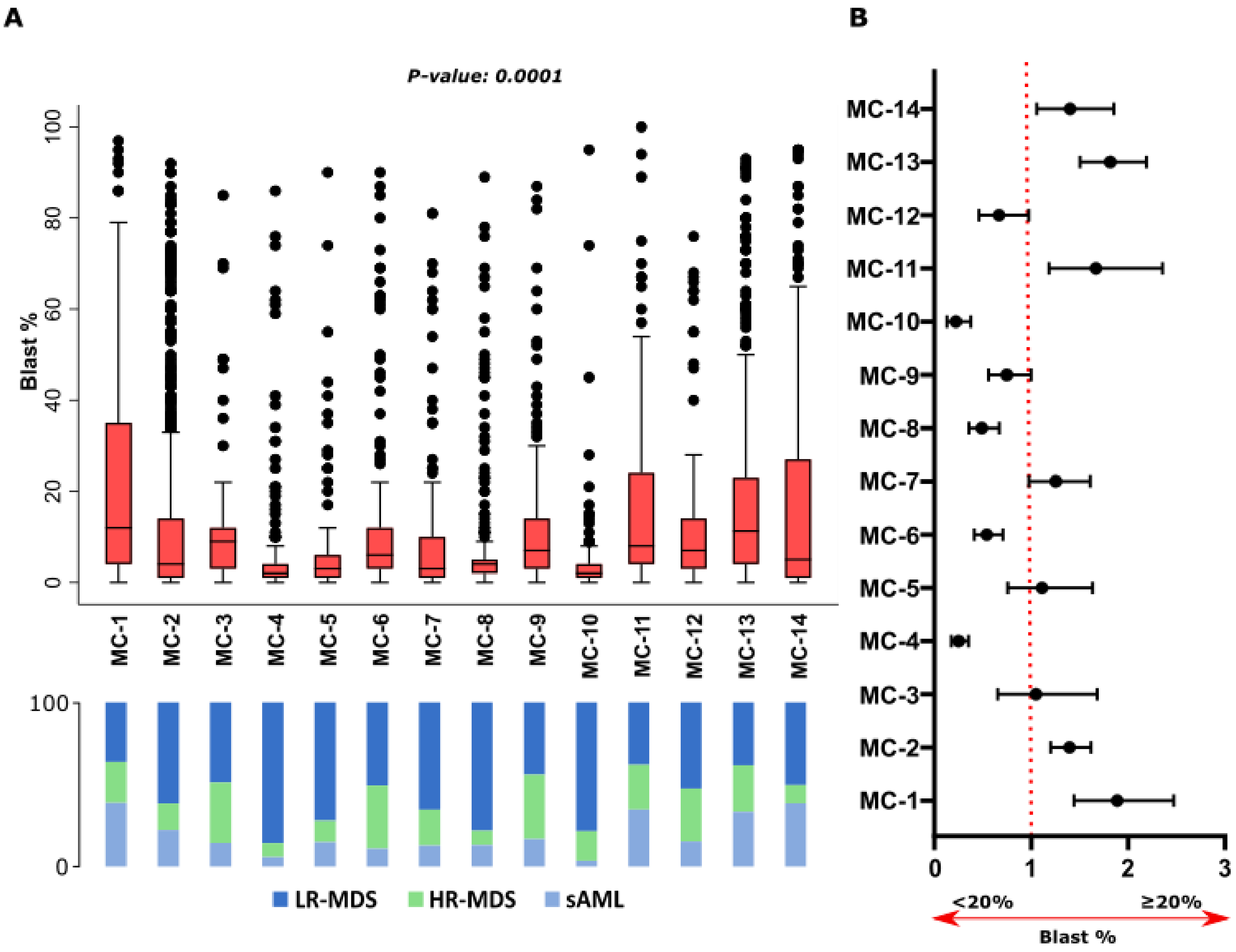

Supplementary Figure-10

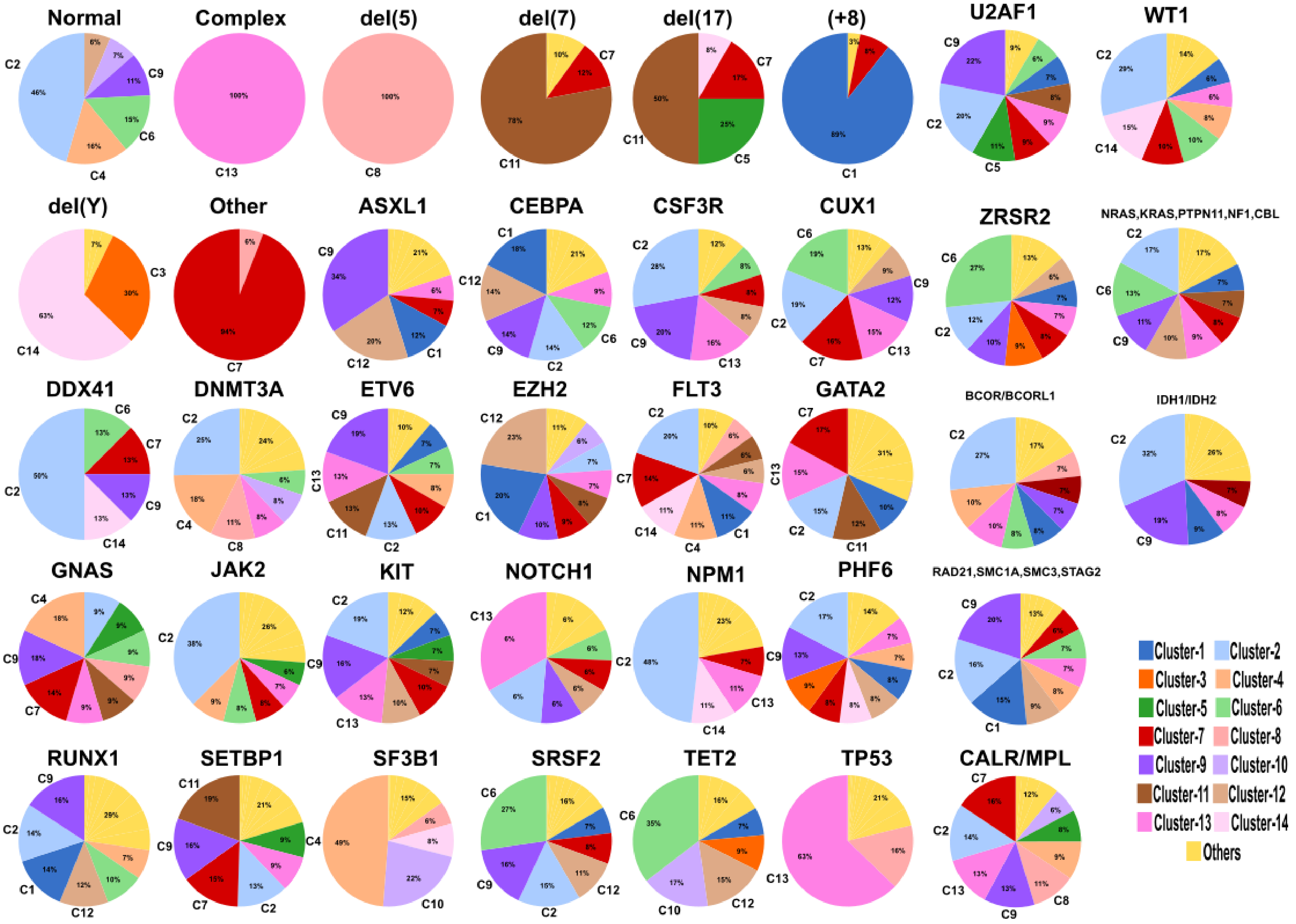

Supplementary Figure-11

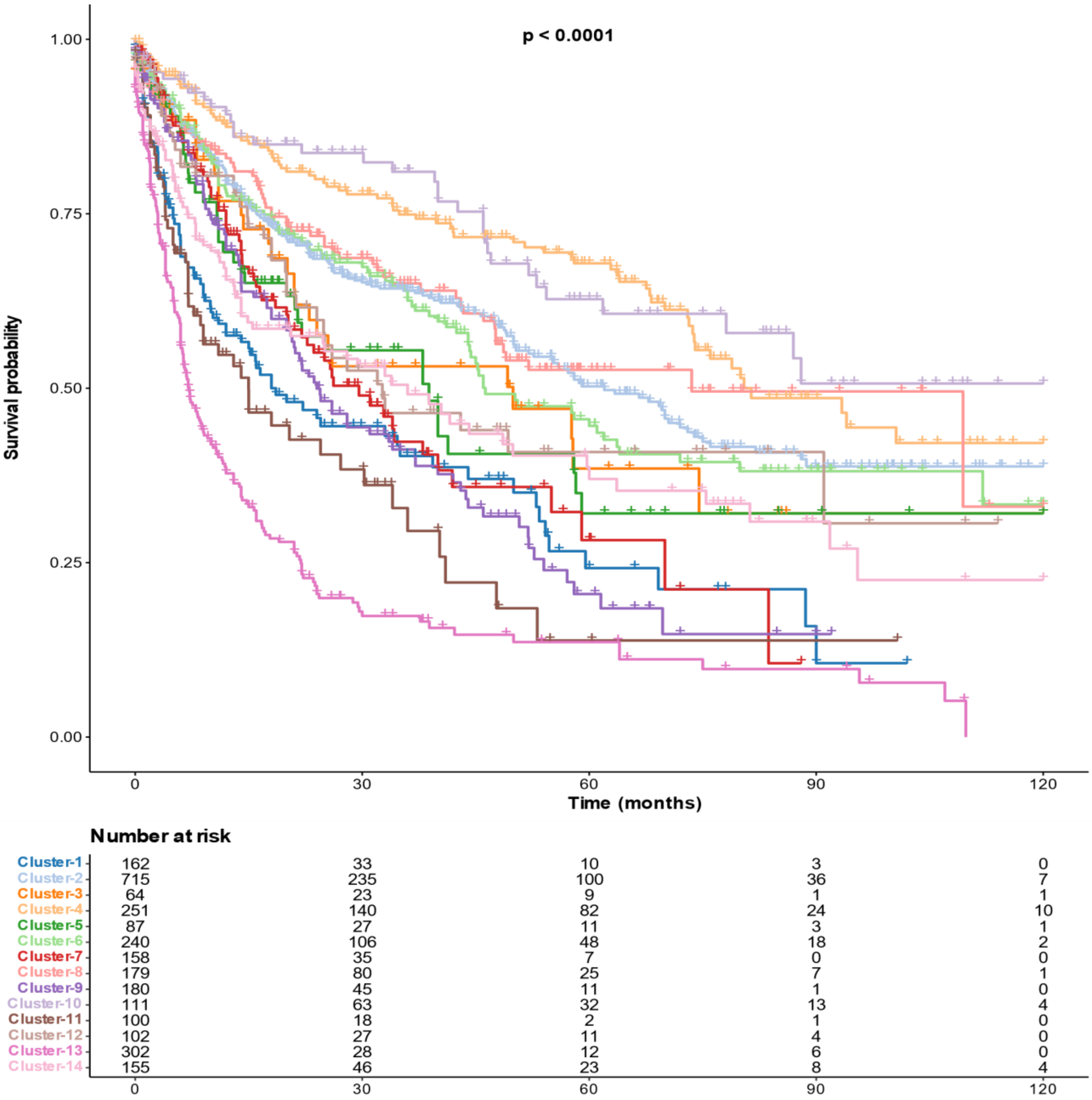

Supplementary Figure-12

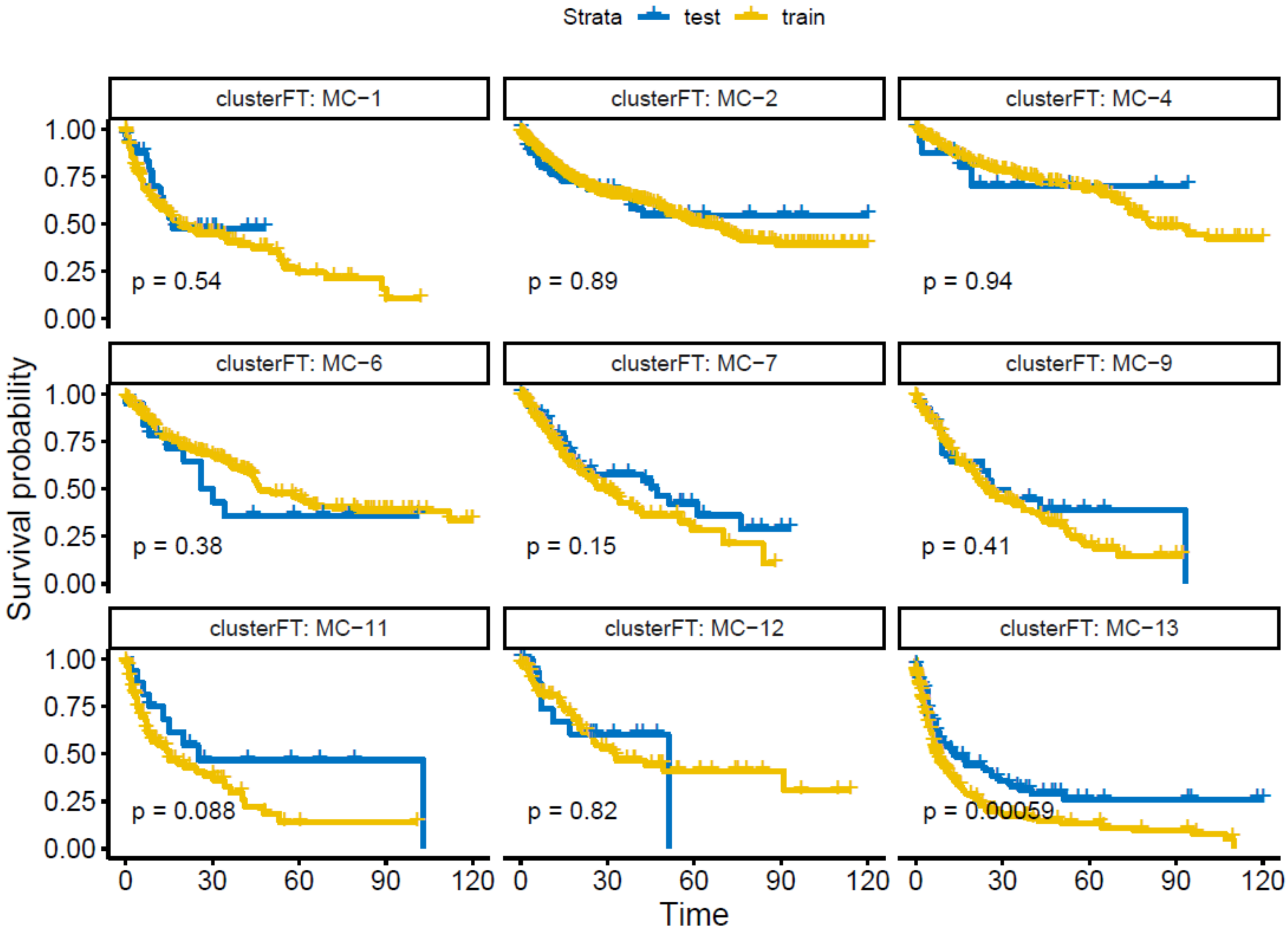

Supplementary Figure-13

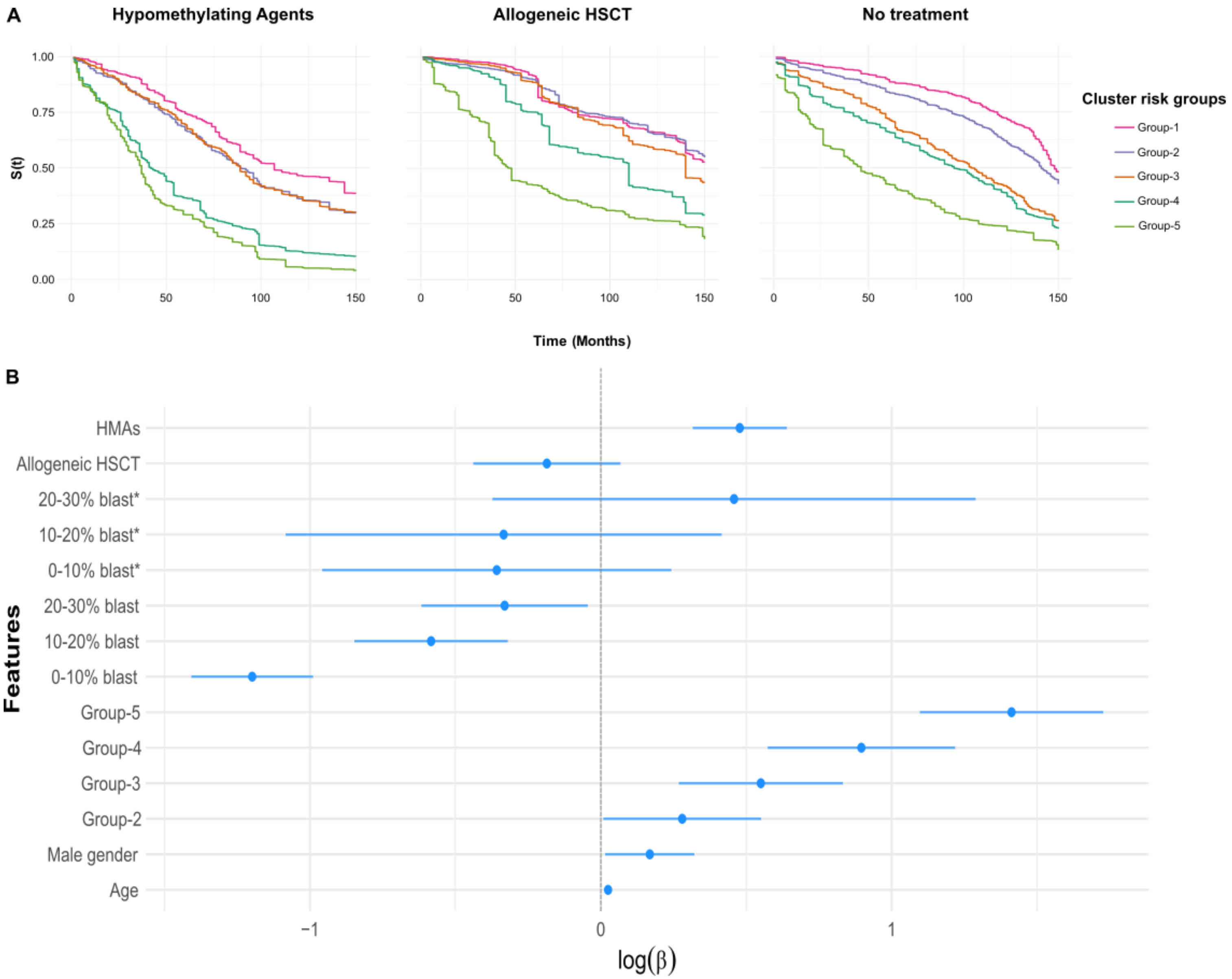

Supplementary Figure-14

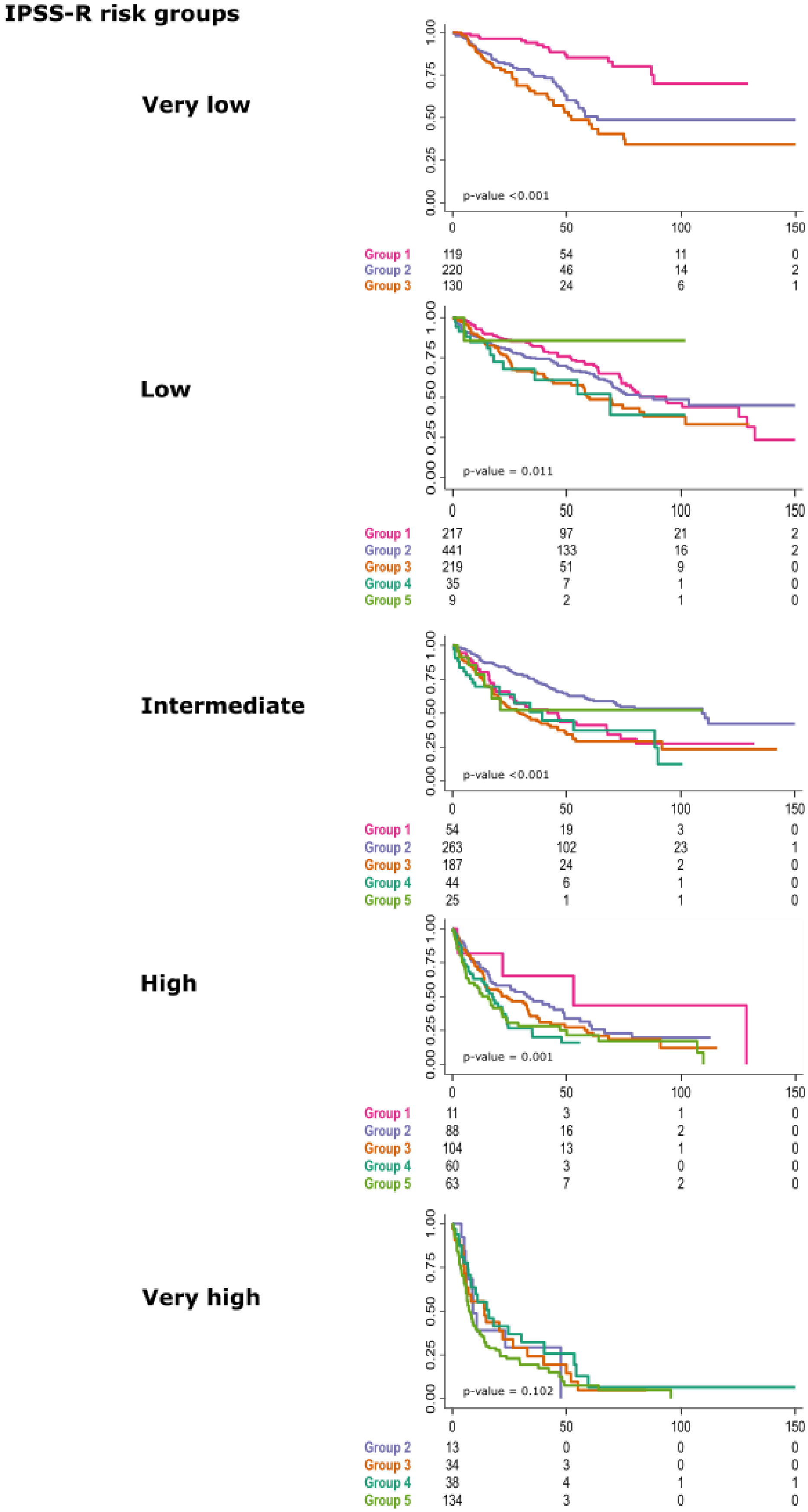

Supplementary Figure-15

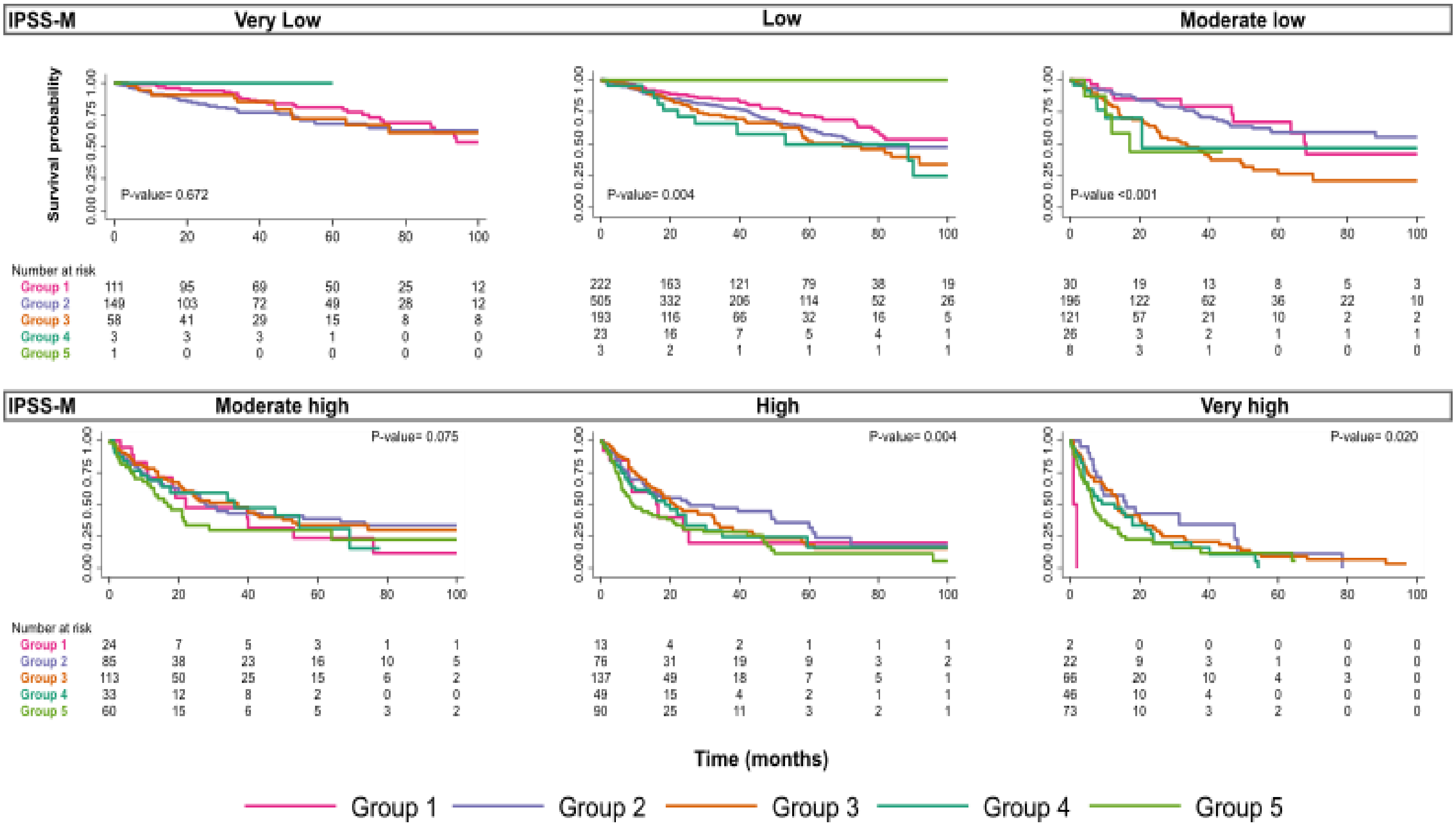

Supplementary Figure-16

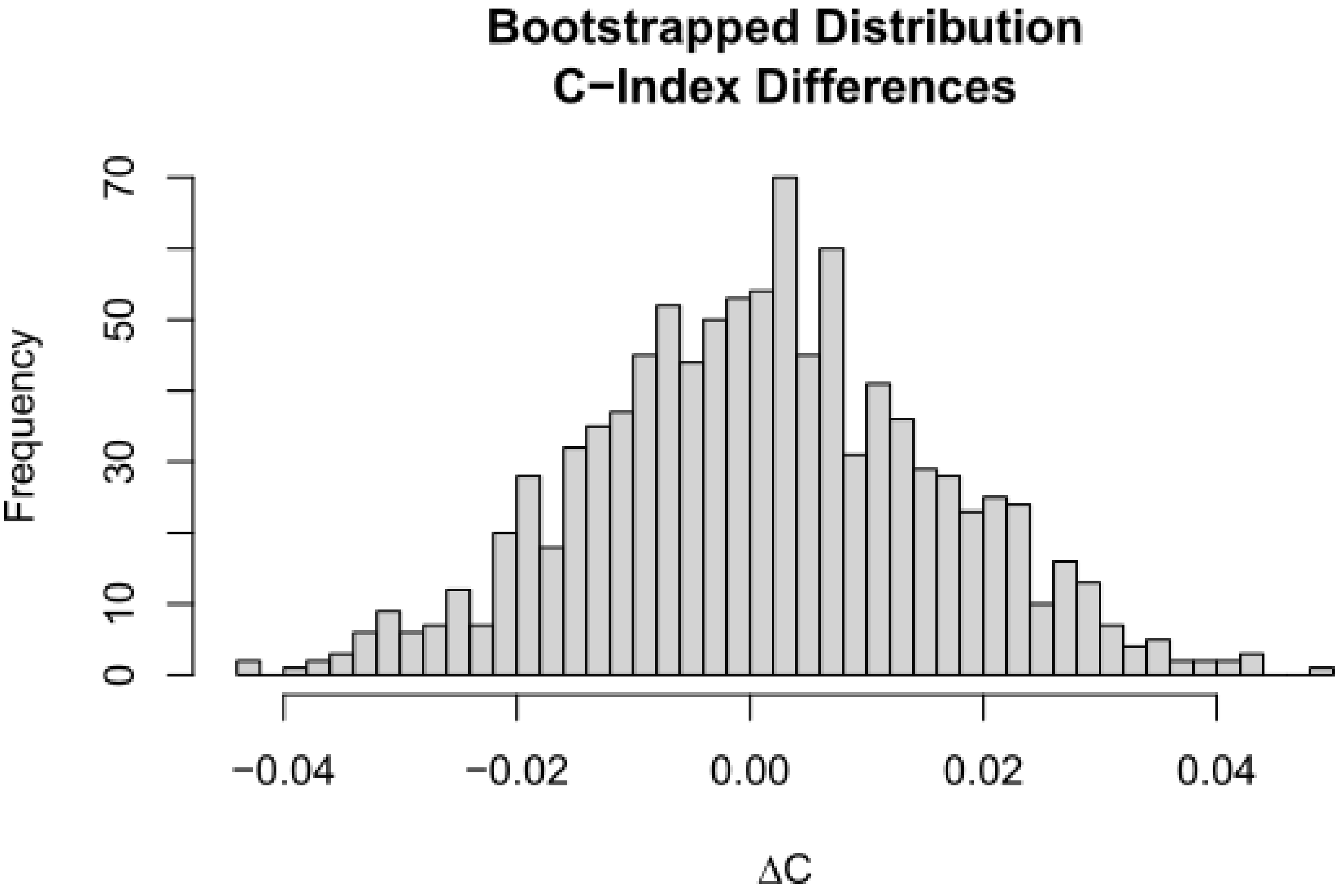

Supplementary Figure-17

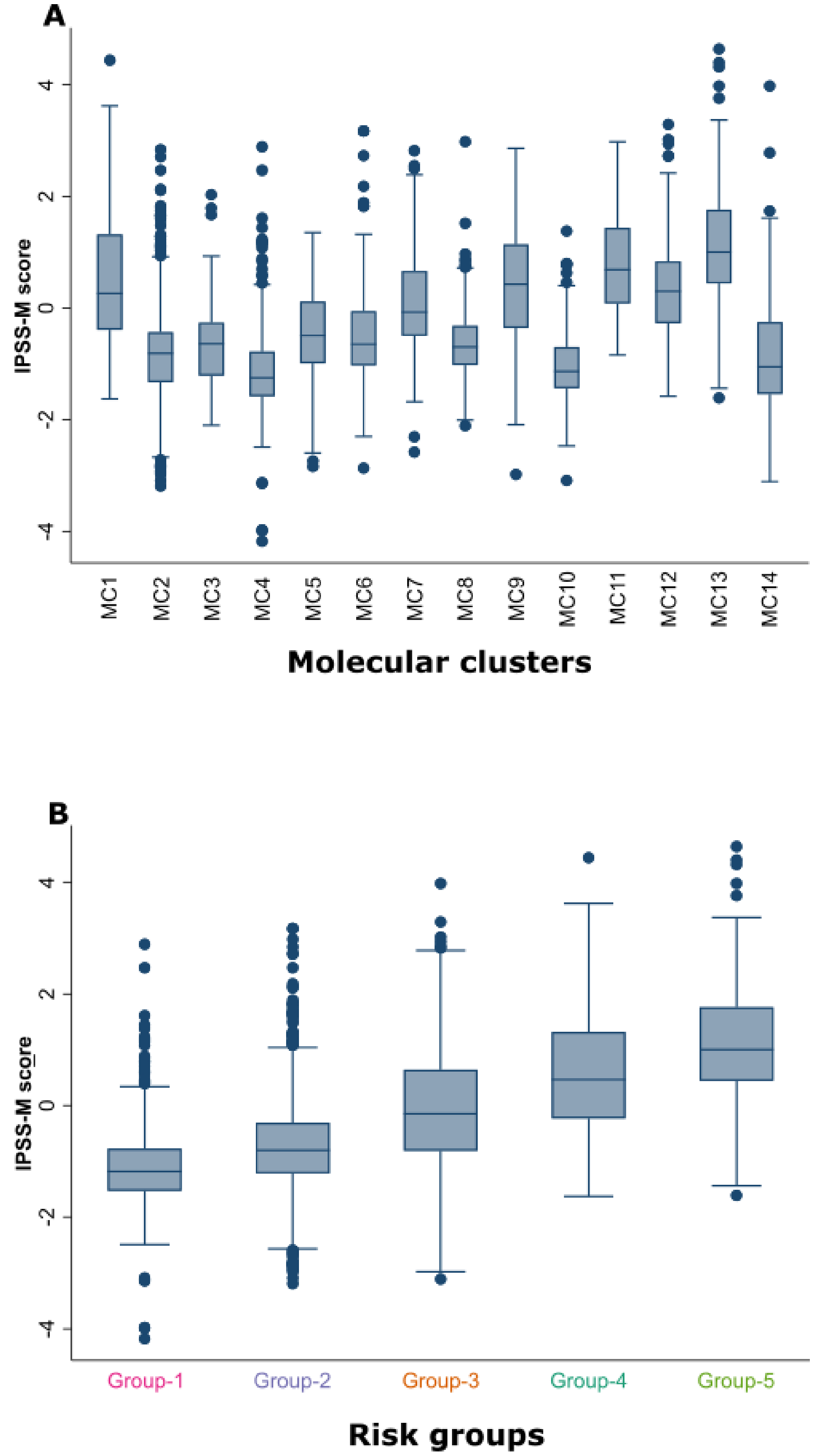

Supplementary figure legends

Supplementary Figure 1. Frequency of total mutations as distributed among our myelodysplastic syndrome (MDS) and secondary acute myeloid leukemia (sAML) cases.

Supplementary Figure 2. A: Histogram bars represent the distribution of molecular hits and cytogenetics abnormalities among LR-MDS, HR-MDS, and sAML patients illustrated by a specific figure color legend. **B:** Heatmap representation of the frequency of molecular mutations and cytogenetic abnormalities per each genomic cluster.

Supplementary Figure 3. Genetic features ordered by 'global importance' measured by mean decrease in accuracy for the random forest classification model. A mean decrease in accuracy≥0.01 was considered significant.

Supplementary Figure 4. Cluster-specific importance of genetic features measured by mean decrease in accuracy for the random forest classification model. A mean decrease in accuracy≥0.01 was considered significant.

Supplementary Figure 5. K-fold cross-validation method for the proposed unsupervised clustering approach. **A:** The figure represents the silhouette values based on the number of the clusters. Total cluster number of 14 was associated with highest silhouette values majority of the folds. **B:** Overlap between the sub-groups (folds) based on the predicted assignments of random-forest classification models generated from each fold separately. More specifically, comparing row j to column k shows the overlap of predictions for cases in fold j quantified by Adjusted Rand Index (ARI) classified by the models trained on fold j and fold k datasets (See supplementary methods for details). **C:** Overlap between the sub-groups (folds) similar to subpanel B where the comparison were done explicitly only on the test datasets. Specifically, comparing row j to column k, ARI shows the overlap of class assignment for samples assigned to remaining folds hence test datasets for models j and k.

Supplementary Figure 6. Clustering based on BayesLCA model as a baseline model comparison. A: Kaplan-Meier estimates showing the overall survival (in months) of cases assigned to different clusters (LCA-C1 to LCA-C5), with the associated 95% CI. **B:** Heatmap showing the overlap between the proposed risk groups (y-axis) and the LCA clusters (x-axis). **C:** Similarly, heatmap showing the overlap between the molecular clusters (y-axis) and the LCA clusters (x-axis) representing relatively good overlap of identified clusters with both models.

Supplementary Figure 7. Clinical characteristics of our molecular clusters (MCs), n=3588 patients**. A:** Bar histogram showing the relative frequencies of male/female in each MC. **B:** Histogram showing the specific morphological diagnosis in each MC. **C:** Boxplot graph presenting the median age of each molecular cluster. Data are represented as box-and-whisker plots (minimum age, 25% percentile, median, 75% percentile, and maximum age respectively for MC1: 20, 67, 74, 80, 100 ; for MC2 18, 59, 69, 76, 94; for MC3 42,69, 75, 82, 99; for MC4 30, 66, 72, 78, 95; for MC5 31, 65, 72, 78, 89; for MC6 23, 68, 74, 78, 95; for MC7 23, 59, 68, 74, 87; for MC8 35, 68, 74, 80, 93; for MC9 20, 64, 72, 77, 90; for MC10 18, 70, 74, 78, 89; for MC11 25, 62, 75, 78, 88; for MC12 47, 69, 75, 80, 88; for MC13 18, 63, 71, 77, 94 ; and for MC14 24, 63, 71, 77, 91. **D:** Boxplot graph presenting the median hemoglobin level in each MC. Data are represented as box-and-whisker plots (minimum hemoglobin, 25% percentile, median, 75% percentile, and maximum hemoglobin respectively for MC1: 5.4, 8.3, 9.4, 10.8, 14.1; for MC2 4.5, 8.8, 10.2, 11.6, 15.9; for MC3 6.1, 8.8, 9.9, 12.4, 14.5; for MC4 2.7, 8.9, 9.8, 10.7, 19.6; for MC5 6.1, 8.9, 9.9, 10.7, 15.6; for MC6 6.0, 10.0, 11.4, 12.9, 16.6; for MC7 4.4, 8.5, 9.8, 11.3, 15.8; for MC8 5.0, 8.4, 9.3, 10.3, 16.5; for MC9 5.8, 8.8, 9.8, 11.2, 17.5; for MC10 6.0, 8.9, 10.0, 10.8, 14.1; for MC11 5.8, 8.0, 9.0, 10.3, 14; for MC12 6.1, 9.0 , 10.2, 11.7, 15.2; for MC13 3.5, 8.0, 9.0, 10.0, 16.1; and for MC14 5.9, 8.2, 9.5, 11.0, 15.5. **E:** Boxplot graph presenting the median white blood cell (WBC) count of each molecular cluster. Data are represented as box-and-whisker plots (minimum WBC, 25% percentile,

median, 75% percentile, and maximum WBC respectively for MC1: 0.4, 2.5, 5.6, 14.2, 125.0; for MC2 0.2, 2.6, 5.1, 11.7, 230.6; for MC3 0.7, 3.5, 7.1, 10.6, 120.1; for MC4 0.1, 4.1, 6.0, 8.5, 226.0; for MC5 0.7, 2.6, 4.6, 9.6, 63.7; for MC6 0.7, 3.1, 5.1, 12.9, 132.0; for MC7 0.3, 2.9, 4.8, 11.9, 84.7; for MC8 0.1, 2.8, 3.0, 5.7, 82.0; for MC9 0.7, 3.2, 6.0, 21.9, 153.1; for MC10 1.9, 3.8, 5.0, 7.0, 162.0; for MC11 0.1, 2.7, 4.2, 19.4, 101.7; for MC12 1.8, 3.3, 9.8, 24.5, 387.5; for MC13 0.2, 1.9, 3.4, 7.6, 228.3; and for MC14 0.7, 2.7, 5.5, 12.7, 197.0. **F:** Boxplot graph presenting the median platelet count in each MC. Data are represented as box-and-whisker plots (minimum platelet, 25% percentile, median, 75% percentile, and maximum platelet respectively for MC1 5.0, 31.5, 87.0, 163.0, 777.0; for MC2 3.0, 54.0, 104.0, 221.0, 964.0 for MC3 3.0, 68.0, 117.5, 160.0, 297.0; for MC4 9.0, 183.5, 307.0, 420.0, 948.0 ; for MC5 6.0, 39.0, 92.0, 171.0, 775.0; for MC6 2.0, 66.0, 100.0, 154.0, 698; for MC7 6.0, 45.5, 110.5, 191.0, 938.0 ; for MC8 3.0, 77.0, 217.0, 321.0, 984.0; for MC9 7.0, 38.0, 95.5, 220.0, 748.0; for MC10 14.0, 146.0, 232.0, 333.0, 963.0; for MC11 1.0, 24.0, 47.5, 130.5, 411.0; for MC12 10.0, 47.0, 83.0, 130.5, 843.0; for MC13 4.0, 30.0, 57.5, 102.0, 593.0; and for MC14 3.0, 40.0, 109.0, 235.0, 869.0. MDS-SLD: myelodysplastic syndrome (MDS) with single lineage dysplasia, MDS-MLD: MDS with multilineage dysplasia, MDS/MPN: MDS/myeloproliferative neoplasm, CMML: chronic myelomonocytic leukemia, MDS-EB1/2: MDS with excess blasts 1/2, sAML: secondary acute myeloid leukemia.

Supplementary Figure 8. Molecular clusters (C) percentage in low-risk myelodysplastic syndrome (LR-MDS), high-risk myelodysplastic syndrome (HR-MDS), and secondary acute myeloid leukemia (sAML) patients. The pie charts demonstrate the percentage of each molecular cluster (C) in different clinical diseases. Each molecular cluster is presented by a specific figure legend color.

Supplementary Figure 9. Bone marrow blast percent (%) per molecular clusters, n=3588 patients. **A:** The box plot represents the distribution of bone marrow blast percent in each molecular cluster (MC), solid lines represent median (upper panel). Data are represented as box-and-whisker plots (minimum blast %, 25% percentile, median, 75% percentile, and maximum blast % respectively for MC1 0%, 4%,12%,37%, 97%; for MC2 0%, 1%, 4%, 14%, 92%; for MC3 0%, 3%, 9%, 12%, 85%; for MC4 0%, 1%, 2%, 4%, 86%; for MC5 0%, 1%, 3%, 6%, 74%; for MC6 0%, 3%, 6%, 12%, 90%; for MC7 0%, 1%,3%, 7%, 35%; for MC8 0%, 2%, 4%, 5%, 89%; for MC9 0%, 3%, 7%, 14%, 87%; for MC10 0%, 1%, 2%, 4%, 95%; for MC11 0%, 4%, 8%, 24%, 94%; for MC12 0%, 3%, 8%, 14%, 76%; for MC13 0%, 3%, 12%, 22%, 93%; and for MC14 0%, 1%, 5%, 25%, 95%. Lower panel represents the distribution of low-risk myelodysplastic syndrome (LR-MDS, bone marrow blast<5%), high-risk MDS (HR-MDS, bone marrow blast ≥5), and secondary acute myeloid leukemia (sAML) within each MC. **B:** Forest plot of BM blast ≥20% odd ratios (one MC vs. others), data are presented as odd ratio +/- 95% confidence interval.

Supplementary Figure 10. Distribution of all the mutations and cytogenetic abnormalities used to build our scheme across molecular clusters (C). The pie charts illustrate the abundance of each molecular cluster (C) with regards to gene mutations and cytogenetic abnormalities. Each molecular cluster is presented by a specific figure legend color. No statistical analysis for comparison was done.

Supplementary Figure 11. Kaplan-Meier analysis showing the overall survival (in months) of cases assigned to different molecular clusters (cluster-1 to cluster-14). Exact p-value associated with the test is 2.26E-77 .

Supplementary Figure 12. Kaplan-Meier analysis showing the overall survival (in months) of cases assigned to different molecular clusters (MC) in the training set (yellow) and the validation cohort (blue). The KM curves for the external validation and training sets showed no difference based on the log-rank test except for MC 13 where comparison of MC-3,5,8,10,14 was hampered by low number of classified cases. No correction for multiple-hypothesis testing was done.

Supplementary Figure 13. A: Non-parametric survival estimation using Random Survival-Forest for different genomic risk groups adjusted for hypomethylating agents (HMAs) treatment, allogeneic hematopoietic stem cell transplant (HSCT), no treatment, age and sex. Survival curves are estimated for a pseudo-patient (male, aged 75 years) showing the effect of molecular clusters adjusting for treatment and other clinical variables. Each risk group is presented by a specific figure legend color. **B:** CoxPH analysis of overall survival (n=1680

patients) according to age, gender, cluster risk groups, bone marrow (BM) blast percent before 25 months and after 25 months (asterisk [*]) to account for the proportionality assumption, HMAs treatment, and allogeneic HSCT. BM is binned into 4 groups from low to high BM percentage where BM-Low is ≤ 10, BM-LowInt ≤ 20, BM-Mid is ≤ 30 and BM-High is > 30. For reference group, BM-High is used. Log-scaled coefficients, standard-error, statistic and p-values are as follows; age (0.025, 0.004, 6.060, 1.361e-09),sexM (0.168, 0.078, 2.150, 3.156e-02),riskInt-Low (0.280, 0.138, 2.022, 4.322e-02),riskInt-High (0.550, 0.144, 3.821, 1.330e-04),riskHigh (0.896, 0.164, 5.452, 4.981e-08),riskPoor (1.412, 0.161, 8.779, 1.644e-18),BMT (-0.186, 0.129, -1.437, 1.508e-01),AZA_DEC (0.478, 0.082, 5.792, 6.944e-09),Bm-Low1 (-1.199, 0.107, -11.231, 2.873e-29),BM-LowInt1 1 (-0.584, 0.135, -4.336, 1.452e-05),BM-Mid1 (-0.331, 0.146, -2.264, 2.357e-02),BM-Low2 (-0.358, 0.306, -1.169, 2.425e-01),BM-LowInt2 (-0.334, 0.383, -0.873, 3.825e-01),BM-Mid2 (0.458, 0.424, 1.081, 2.799e-01).

Supplementary Figure 14. Kaplan-Meier analysis showing the overall survival (in months) of cases assigned to different molecular risk groups (Group 1-5) among different Revised International Prognostic Scoring System (IPSS-R) risk groups. Statistically significant difference of log-Rank test is indicated by the p-value (very low: 2.682E-07, low: 0.01099981, intermediate: 0 .00025526, high: 0 .00120386, very high: 0.10195813).

Supplementary Figure 15. Kaplan-Meier analysis showing the overall survival (in months) of cases assigned to different molecular risk groups (Group 1-5) among different Molecular International Prognostic Scoring System (IPSS-M) risk groups. Statistically significant difference of log-Rank test is indicated by the p-value (very low: 0.67240292, low: 0.00430145, moderate low: 0 .00001269, moderate high: 0 .07487812, high: 0.0041087, very high: 0 .01954389).

Supplementary Figure 16. Histogram showing the bootstrap distribution of the Harrell's C-index differences (delta [Δ] C) between the proposed clusters and IPSS-M adjusting for clinical variables age, sex, log(BM), log(PLT) and HB.

Supplementary Figure 17. Molecular International Prognostic Scoring System (IPSS-M) scores, n=2271 patients. Boxplot showing the median IPSS-M scores for each molecular cluster (MC). Data are represented as box-and-whisker plots (minimum IPSS-M score, 25% percentile, median, 75% percentile, and maximum IPSS-M score respectively. Similarly, quantiles for molecular clusters (%2.5, %25, %50, %75, %97.5) for **A:** MC 1 (-1.5025,-0.3875,0.265,1.155,2.44875),MC 2 (-2.5835,-1.33,-0.81,-0.43,1.28),MC 3 (-1.90375,-0.91,-0.61,-0.2375,1.751),MC 4 (-2.4705,-1.6,-1.26,-0.845,1.0815),MC 5 (-2.44,-0.99,-0.49,0.17,1.17),MC 6 (-2.0325,-1.03,-0.69,-0.085,1.30875),MC 7 (-1.28275,-0.51,-0.07,0.6025,2.27925),MC 8 (-1.81125,-1.1025,-0.735,-0.32,0.735),MC 9 (-1.4615,-0.46,0.39,1.085,2.651),MC 10 (-1.9655,-1.385,-1.08,-0.585,0.678999999999999),MC 11 (-0.8045,0.06,0.63,1.29,2.628),MC 12 (-1.4305,-0.27,0.285,0.77,2.8905),MC 13 (-0.1995,0.44,1.09,1.88,3.2985),MC 14 (-2.2925,-1.53,-1.03,-0.145,1.475) and for risk groups **B:**. Group 1 (-2.2945, -1.525, -1.18, -0.765,0.827), Group 2 (-2.35375, -1.22, -0.8, -0.3225,1.28), Group 3 (-2.02, -0.8, -0.13,0.61,2.387), Group 4 (-1.306, -0.22,0.45,1.24,2.47), Group 5 (-0.1995,0.44,1.09,1.88,3.2985)

Supplementary Methods

Genetic studies

For the data collected at Cleveland Clinic, whole-exome sequencing (WES) was performed and paired disease and germline DNA of purified CD3+ lymphocytes were used. Whole-exome capture was accomplished by hybridizing sonicated genomic DNAs to a bait cDNA library synthesized on magnetic beads (SureSelect Human All Exon 50Mb or V4 kit, Agilent Technologies). Captured targets were sequenced using a HiSeq 2000 (Illumina) and standard protocols for 100-bp paired-end reads. Reads were aligned to the human genome (hg19) by a Burrows-Wheeler aligner (http://bio-bwa.sourceforge.net/) using a GATK pipeline that extracted candidate

variants/polymorphisms to reduce sequencing errors. Data were validated using targeted sequencing. Targeted sequencing was performed using a TruSeq Custom Amplicon kit (Illumina); a panel of 40 genes was interrogated (Supplementary Table-2). Sequencing libraries were generated according to an Illumina paired-end library protocol. The enriched targets were sequenced using a HiSeq 2000 or MiSeq (Illumina), at 862x coverage. Variants were annotated using Annovar and filtered by removing: i) synonymous single nucleotide variants; ii) variants only present in 140 unidirectional reads; and iii) variants in repetitive genomic regions. Variants with minimum depth less than 20 or number of high-quality reads less than 5 were filtered out. A bio-analytic pipeline developed in-house[1-3] deidentified somatic mutations using sequences derived from controls and mutational databases such as dbSNP138, 1000 Genomes or ESP 6500 database, and Exome Aggregation Consortium (ExAC). Variant allelic frequencies (VAFs) were adjusted according to zygosity and copy number based on conventional metaphase karyotyping and/or single nucleotide polymorphism array results. Patients from the MLL cohort were investigated by NGS using different methods and gene panels as previously described[1,2,4]. The gene sequencing methods of publicly-shared patients were previously described[5-8].

Conventional cytogenetics

Metaphase cytogenetics was performed on bone marrow (BM) aspirates. The median number of metaphases analyzed was 20. Chromosomal preparation was performed on G-banded metaphase cells using standard techniques, and karyotypes were described all the patients according to the International System for Human Cytogenetic Nomenclature[9,10].

Statistical methods

Fisher's exact test and Chi-square test were used to compare categorical variables. Mann–Whitney U test/ Wilcoxon rank-sum test were used for continuous variables. All p- values were two-sided; those less than 0.05 were considered statistically significant. All statistical computations were performed using R 3.6.2 (www.r-project.org) and Prism (GraphPad). To assess prognostic differences among the identified clusters, pairwise survival analysis using Kaplan Meier estimator and log-rank test was performed.

Autoencoder

Autoencoders are neural-network architectures, which can be designed to generate efficient compressed/low dimensional representations of the input data. Here, we used a single-layer Autoencoder with shared layer between encoder and decoder to generate low-dimensional representations of binary mutation profiles of MDS samples. Specifically, mutations and cytogenetic alterations were encoded as binary features based on presence or absence. With a single shared layer, autoencoders can learn to capture the principal component space without orthogonality constraint, hence a proxy for binary-PCA analysis. We used TensorFlow framework to optimize via Adam optimizer and learning rate and batch size set to 1e-4 and 32 respectively. l1 and l2 regularization parameters are set to 0.1 as well to prevent possible overfitting[11,12].

Gaussian mixture model

Gaussian-mixture models (GMM) are model-based clustering methods where observations are separated into components/clusters representing gaussian distributions parameterized by different μ and σ. We used scikit-learn package to fit GMM with expectation-maximization over increasing number of components and used Bayesian Information Criterion (BIC) to select the number of clusters. Over 100 iterations with random sub-sampling of binary observation vectors of mutation profiles, we first generated a low dimensional embedding of the sub-sampled data and used GMM to cluster the observations, where the number of GMM components was selected using Bayesian Information Criterion (BIC). Keeping track of co-clustering of observations at each iteration, we generated a consensus-matrix representing the frequency of clustering observations in the same cluster. The generated consensus-matrix was further clustered using hierarchical-clustering with Ward's criteria to create the final cluster assignments using Silhouette value to select the number of clusters[13].

Unsupervised clustering

Coupling Autoencoders and GMM, we generated unsupervised clusters of MDS cases via Consensus approach. Over 100 iterations with random sub-sampling of data, we embedded the observations using the single layer autoencoder and clustered using the gaussian-mixture model. Keeping track of co-clustering of observations we generated a consensus-matrix. Finally, the consensus-matrix is clustered using hierarchical clustering with Ward's criteria and Silhouette value to select the number of clusters to generate the final cluster assignments for all cases as described in more detail above.

Classification of new data

Since our unsupervised clustering approach is a 'distance' based approach quantified by the frequency of co-occurrence, new datasets cannot be readily assigned to individual clusters. For this purpose, we utilized Random Forests via R package randomForest in order to generate a multi-class classification model using binary mutation profiles as input and molecular clusters as classes to be predicted. Hyperparameter tuning was done via cross-validation. Trained model was used to both classify internal and external validation cohorts and is also available as a web based open-source tool. In order to include the risk stratification to the assigned molecular clusters, we generated a random-forest classifier using the identified risk groups as well. We then followed a similar approach for hyperparameter tuning. Furthermore, our risk stratification grouping has been added to the web tool to help with the clinical application of our model.

Validation

The model was internally and externally validated. Internal validation was performed by dividing the whole cohort into training (80%), test (20%) sets and evaluating on the test set. Survival analysis was then performed on the remaining 20% test set separately for internal validation. Furthermore, since we do not have 'true' class labels, in order to evaluate how robust the clustering across different folds is, we used each 20% data set (fold) as a training set and generated 5 distinct models and trained on each fold separately. The comparisons across the models are hence done in an asymmetric fashion. Specifically, comparing model 2 to model 3 for instance, we are comparing the predictions of model 2 on the fold 2 data (which is the training set for model 2) and the predictions of model 3 on the fold 2 data (which is now a test set for model 3). Hence, model 2 predictions are now a proxy for a 'true' class label. In contrast, when comparing model 3 to model 2, we are comparing predictions of model 3 on fold 3 data with predictions of model 2 on fold 3 data. Nevertheless, we also conducted an explicit comparison as well using unseen data for both models. So, the comparisons are made between predictions of model 2 done on folds 1,4, and 5 datasets and predictions of model 3 done on folds 1,4, and 5 datasets (keeping fold 2 and fold 3 datasets aside), External validation was conducted on an independent cohort of MDS and sAML patients from the University of Texas Southwestern and the Wayne State University (Supplementary Table 1).

Supplementary notes

Examples of molecular associations in our MCs:

Example 1

SF3B1 mutations were enriched in MC4, MC10, and MC14 and associated with *TET2* (100%) mutations in MC10, indicating functional relationships. Notably, this group was distinct from MC14 in which *SF3B1* mutations were grouped with delY and *SRSF2* mutations. The diverse pathobiological derivation of these groups was also illustrated by their significant survival differences. Other splicing factor mutations were instead functionally

distinctive and were assigned to different genomic groups. Indeed, *SRSF2* mutations were enriched in MC9 and MC12 and associated with *ASXL1* mutations and normal karyotype, but MC12 cases had additional *TET2* mutations. A similar principle seemed to apply to *U2AF1* mutations, abundant in MC5. These results argue that the genomic sub-classification of splicing factor mutations is strongly dependent on the presence/absence of other unique correlating cofounders affecting the functionality and biology of the distinct cluster.

Example 2

Traditionally, epigenetic modulator mutations have been grouped together for classification purposes, often ignoring their distinct or even often opposite function[5,14,15]. In contrast, our ML-derived model highlights these functional differences, underscoring intertwining relationships across different molecular pathways of leukemogenesis. For instance, we found that *IDH1/IDH2* mutations were mainly abundant in two MCs (MC2 and MC9), which had discrete survival differences and a significantly higher percentage of *STAG2* and *IDH1/IDH2* mutations in MC9 vs. MC2, in which they coincided with more frequent *RAS* and *DNMT3A* hits. Similarly, the functional effect of *DNMT3A/SF3B1* co-mutations was reflected by a better OS of MC4 and MC10 (Low-Risk group). This phenomenon can be functionally explained by the relative mitigation effects of *SF3B1* mutation on *DNMT3A* clones[16,17]. Because *DNMT3A* and *TET2* have different biological and possibly opposite functions, mutations in these epigenetic regulators belong to distinct functional pathways and separate MCs. Both *EZH2* and *UTX* possess opposite *H3K27* methylation effects and thus their mutations clustered separately and associated/substituted with/for distinct cluster-defining hits. Finally, *RUNX1* mutations were abundant in MC9, MC12 and MC1 and were significantly associated with either *ASXL1* and *SRSF2* mutations as previously reported[18].

Example 3

While our results reaffirm previous studies regarding the poor survival outcome associated with complex cytogenetics and *TP53* mutations[5,19-21], the impact of *TP53* allelic configuration on pathology and prognosis was also reflected in our molecular clustering, e.g., *TP53* mutations in MC8 were mostly monoallelic (70%), explaining the better outcomes as compared to other MCs with *TP53* in biallelic configuration. In addition, mutually exclusive *PPM1D* mutations coincided with *TP53* clusters (MC13 and MC8), pointing towards to the known similar pathogenic pathway[22-24].

References

1. Meggendorfer M, Haferlach C, Kern W, et al: Molecular analysis of myelodysplastic syndrome with isolated deletion of the long arm of chromosome 5 reveals a specific spectrum of molecular mutations with prognostic impact: a study on 123 patients and 27 genes. Haematologica 102:1502-1510, 2017
2. Delic S, Rose D, Kern W, et al: Application of an NGS-based 28-gene panel in myeloproliferative neoplasms reveals distinct mutation patterns in essential thrombocythaemia, primary myelofibrosis and polycythaemia vera. Br J Haematol 175:419-426, 2016
3. Haferlach T, Nagata Y, Grossmann V, et al: Landscape of genetic lesions in 944 patients with myelodysplastic syndromes. Leukemia 28:241-7, 2014
4. Palomo L, Meggendorfer M, Hutter S, et al: Molecular landscape and clonal architecture of adult myelodysplastic/myeloproliferative neoplasms. Blood 136:1851-1862, 2020
5. Bersanelli M, Travaglino E, Meggendorfer M, et al: Classification and Personalized Prognostic Assessment on the Basis of Clinical and Genomic Features in Myelodysplastic Syndromes. J Clin Oncol 39:1223-1233, 2021
6. Nagata Y, Zhao R, Awada H, et al: Machine learning demonstrates that somatic mutations imprint invariant morphologic features in myelodysplastic syndromes. Blood 136:2249-2262, 2020
7. Radakovich N, Nagy M, Nazha A: Machine learning in haematological malignancies. Lancet Haematol 7:e541-e550, 2020

8. Tyner JW, Tognon CE, Bottomly D, et al: Functional genomic landscape of acute myeloid leukaemia. Nature 562:526-531, 2018

9. Gonzalez Garcia JR, Meza-Espinoza JP: Use of the International System for Human Cytogenetic Nomenclature (ISCN). Blood 108:3952-3; author reply 3953, 2006

10. Liehr T: International System for Human Cytogenetic or Cytogenomic Nomenclature (ISCN): Some Thoughts. Cytogenet Genome Res 161:223-224, 2021

11. Baldi AP: Autoencoders, Unsupervised Learning, and Deep Architectures. Presented at the Proceedings of ICML Workshop on Unsupervised and Transfer Learning, 2012

12. Hinton GE, Salakhutdinov RR: Reducing the dimensionality of data with neural networks. Science 313:504-7, 2006

13. Fabian Pedregosa GV, Alexandre Gramfort, Vincent Michel, Bertrand Thirion, Olivier Grisel, Mathieu Blondel, Peter Prettenhofer, Ron Weiss, Vincent Dubourg, Jake Vanderplas, Alexandre Passos, David Cournapeau, Matthieu Brucher, Matthieu Perrot, Édouard Duchesnay: Scikit-learn: Machine Learning in Python. Journal of Machine Learning Research 12(85)::2825−2830, 2011

14. Reilly B, Tanaka TN, Diep D, et al: DNA methylation identifies genetically and prognostically distinct subtypes of myelodysplastic syndromes. Blood Adv 3:2845-2858, 2019

15. Venney D, Mohd-Sarip A, Mills KI: The Impact of Epigenetic Modifications in Myeloid Malignancies. Int J Mol Sci 22, 2021

16. Lin ME, Hou HA, Tsai CH, et al: Dynamics of DNMT3A mutation and prognostic relevance in patients with primary myelodysplastic syndrome. Clin Epigenetics 10:42, 2018

17. Song J, Hussaini M, Qin D, et al: Comparison of SF3B1/DNMT3A Comutations With DNMT3A or SF3B1 Mutation Alone in Myelodysplastic Syndrome and Clonal Cytopenia of Undetermined Significance. Am J Clin Pathol 154:48-56, 2020

18. Gaidzik VI, Teleanu V, Papaemmanuil E, et al: RUNX1 mutations in acute myeloid leukemia are associated with distinct clinico-pathologic and genetic features. Leukemia 30:2282, 2016

19. Bahaj W, Gurnari C, Kewan T, et al: The Drive to Acquire Biallelic Hits Inversely Correlates with the Functional Impact of the Primary TP53 Lesion: The Complexity of TP53 Role Assessment. Blood 138:3322, 2021

20. Bernard E, Nannya Y, Hasserjian RP, et al: Implications of TP53 allelic state for genome stability, clinical presentation and outcomes in myelodysplastic syndromes. Nat Med 26:1549-1556, 2020

21. Grob T, Al Hinai AS, Sanders MA, et al: Molecular Characterization of Mutant Tp53 Acute Myeloid Leukemia and High-Risk Myelodysplastic Syndrome. Blood, 2022

22. Kim B, Won D, Lee ST, et al: Somatic mosaic truncating mutations of PPM1D in blood can result from expansion of a mutant clone under selective pressure of chemotherapy. PLoS One 14:e0217521, 2019

23. Kindler T: CHIPing out PPM1D-mutant hematopoiesis. Blood 132:1087-1088, 2018

24. Singh A, Mencia-Trinchant N, Griffiths EA, et al: Mutant PPM1D- and TP53-Driven Hematopoiesis Populates the Hematopoietic Compartment in Response to Peptide Receptor Radionuclide Therapy. JCO Precis Oncol 6:e2100309, 2022
