# Supplementary material for: Molecular patterns identify distinct subclasses of myeloid neoplasia
Source: Nat Commun. 2023 May 30;14:3136. doi: 10.1038/s41467-023-38515-4 (PMC10229666; doi:10.1038/s41467-023-38515-4)
Supplement: Supplementary file 2 — Reporting Summary [file 41467_2023_38515_MOESM2_ESM.pdf]

nature portfolio

Corresponding author(s): Jaroslaw P. Maciejewski and Valeria Visconte

Last updated by author(s): 4/1/2023

Reporting Summary

Nature Portfolio wishes to improve the reproducibility of the work that we publish. This form provides structure for consistency and transparency in reporting. For further information on Nature Portfolio policies, see our Editorial Policies and the Editorial Policy Checklist.

Statistics

For all statistical analyses, confirm that the following items are present in the figure legend, table legend, main text, or Methods section.

n/a	Confirmed	
☐	☒	The exact sample size (*n*) for each experimental group/condition, given as a discrete number and unit of measurement
☐	☒	A statement on whether measurements were taken from distinct samples or whether the same sample was measured repeatedly
☐	☒	The statistical test(s) used AND whether they are one- or two-sided<br>*Only common tests should be described solely by name; describe more complex techniques in the Methods section.*
☐	☒	A description of all covariates tested
☐	☒	A description of any assumptions or corrections, such as tests of normality and adjustment for multiple comparisons
☐	☒	A full description of the statistical parameters including central tendency (e.g. means) or other basic estimates (e.g. regression coefficient) AND variation (e.g. standard deviation) or associated estimates of uncertainty (e.g. confidence intervals)
☐	☒	For null hypothesis testing, the test statistic (e.g. *F*, *t*, *r*) with confidence intervals, effect sizes, degrees of freedom and *P* value noted<br>*Give P values as exact values whenever suitable.*
☒	☐	For Bayesian analysis, information on the choice of priors and Markov chain Monte Carlo settings
☒	☐	For hierarchical and complex designs, identification of the appropriate level for tests and full reporting of outcomes
☒	☐	Estimates of effect sizes (e.g. Cohen's *d*, Pearson's *r*), indicating how they were calculated

Our web collection on statistics for biologists contains articles on many of the points above.

Software and code

Policy information about availability of computer code

Data collection	No software was used for data collection
Data analysis	Statistical analysis done using R version 4.1.3. R software random forest package v1.0 (https://github.com/ardadurmaz/mds_latent) is openly accessible

For manuscripts utilizing custom algorithms or software that are central to the research but not yet described in published literature, software must be made available to editors and reviewers. We strongly encourage code deposition in a community repository (e.g. GitHub). See the Nature Portfolio guidelines for submitting code & software for further information.

Data

Policy information about availability of data

All manuscripts must include a data availability statement. This statement should provide the following information, where applicable:

- Accession codes, unique identifiers, or web links for publicly available datasets
- A description of any restrictions on data availability
- For clinical datasets or third party data, please ensure that the statement adheres to our policy

All the data (molecular and clinical) used to support our results are available in the article. Public cohort (The BEAT AML Master Trial) is available in the following article:
Burd, A., et al., Precision medicine treatment in acute myeloid leukemia using prospective genomic profiling: feasibility and preliminary efficacy of the Beat AML

Srudy, Nature. 2018 Oct;562(7728):526-531. doi: 10.1038/s41586-018-0623-z

All data used to generate the results of this study, processed data and scripts to ensure reproducibility can be found at https://github.com/ardadurmaz/mds_latent. Additional information and raw data are immediately available upon request to maciejj@ccf.org. All other information are provided in the Supplementary Information/ Tables.

Human research participants

Policy information about studies involving human research participants and Sex and Gender in Research.

Reporting on sex and gender	Our cohort reflects the occurrence of MDS in both genders. Both genders were included.
Population characteristics	We combined cytogenetic and molecular features from a multicenter cohort of 3588 MDS and secondary AML patients to generate a molecular-based scheme using machine learning methods and then externally validated the model on 412 patients. Characteristics are found in Table-1
Recruitment	We assembled a large cohort of patients diagnosed with MDS and sAML to generate a comprehensive genomic data set. Patient data from the Cleveland Clinic ([CC], n=1627), The Munich Leukemia Laboratory ([MLL], n=1275), and publicly available data sets (The BEAT AML master trial and The EuroMDS cohort Patients, n=686) was retrospectively collected. Samples were unique and not longitudinally assessed. Only patients with NGS molecular testing were included in the study. Patients with missing molecular testing or clinical information were excluded.
Ethics oversight	Specimens were collected after receiving written informed consent in accordance with the Declaration of Helsinki and in agreement with IRBs of the participating institutions. The study was approved by the Cleveland Clinic IRB.

Note that full information on the approval of the study protocol must also be provided in the manuscript.

Field-specific reporting

Please select the one below that is the best fit for your research. If you are not sure, read the appropriate sections before making your selection.

[x] Life sciences [ ] Behavioural & social sciences [ ] Ecological, evolutionary & environmental sciences

For a reference copy of the document with all sections, see nature.com/documents/nr-reporting-summary-flat.pdf

Life sciences study design

All studies must disclose on these points even when the disclosure is negative.

Sample size	A total of 3,588 myeloid neoplasms was collected. No statistical methods used to determine sample size. Sample size was comparable to the published cohorts in the literature. Development of the model and its performance was assessed in multiple folds until satisfying performance reached. Sample sizes in the external cohorts were determined by data availability.
Data exclusions	All patients had molecular data available.
Replication	The genomic data of our cohort was consistent with previous finding in similar populations. Same specimen source was used for bulk NGS. We provide source data files for the replication of our model and each figure provided in the manuscript.
Randomization	Splits for training and testing cohorts for internal validation were performed on case-level randomization
Blinding	Co-authors were not blinded during the annotation process since we included heterogeneous spectrum of myeloid neoplasms. Randomization to training and testing sets was blinded.

Reporting for specific materials, systems and methods

We require information from authors about some types of materials, experimental systems and methods used in many studies. Here, indicate whether each material, system or method listed is relevant to your study. If you are not sure if a list item applies to your research, read the appropriate section before selecting a response.

Materials & experimental systems

n/a	Involved in the study
☒	☐ Antibodies
☒	☐ Eukaryotic cell lines
☒	☐ Palaeontology and archaeology
☒	☐ Animals and other organisms
☒	☐ Clinical data
☒	☐ Dual use research of concern

Methods

n/a	Involved in the study
☒	☐ ChIP-seq
☒	☐ Flow cytometry
☒	☐ MRI-based neuroimaging
